# The isoflavone genistein selectively stimulates major satellite repeat transcription in mouse heterochromatin

**DOI:** 10.1186/s13072-025-00623-4

**Published:** 2025-08-25

**Authors:** Thomas Fuhrmann, Nicholas Shukeir, Reagan W. Ching, Galina Erikson, Yuan Dou, Zoe Sawitzki, Megumi Onishi-Seebacher, Carmen Galan, Thomas Jenuwein

**Affiliations:** 1 Max Planck Institute of Immunobiology and Epigenetics (MPI-IE), Freiburg, Germany; 2 Faculty of Biology, International Max Planck Research School for Molecular and Cellular Biology (IMPRS-MCB) and University of Freiburg, Freiburg, Germany; 3 Present Address: Integrated DNA Technologies, Louvain, Belgium; 4 Present Address: Gold Standard Diagnostics, Madrid, Spain

**Keywords:** Genistein, Heterochromatin, Major satellite repeats, DNA topoisomerases, Growth inhibition, Anticancer

## Abstract

Mouse heterochromatin is characterized by A/T-rich, 234 bp DNA repeat arrays, called major satellite repeats (MSR). We investigated MSR expression in response to a variety of stress conditions by using small molecule compounds. We identified the isoflavone genistein to selectively stimulate MSR transcription, but not that of other DNA repeat elements. Genistein is a natural compound that is frequently used in dietary supplements and has been associated with reducing cancer risk. A 24 h exposure of mouse embryonic fibroblasts (MEF) to genistein results in a more than 100-fold induction of MSR transcripts. This up-regulation depends on the activity of RNA polymerase II and requires a cycling G1 cell population. Blocking the cell cycle at the G2/M stage significantly attenuates genistein-mediated stimulation of MSR transcription. Mechanistically, DNA topoisomerase poisons phenocopy the genistein-dependent up-regulation of MSR expression. Together, these data suggest that MSR transcriptional response is guided by an altered topology of the underlying A/T-rich MSR DNA repeat arrays and reveal a novel function for genistein that may contribute to the anticancer properties of this natural compound.

**Supplementary Information:**

The online version contains supplementary material available at 10.1186/s13072-025-00623-4.

## Background

Heterochromatin has important functions in the structural and functional organization of eukaryotic chromatin [1]. In mouse cells, heterochromatin is characterized by A/T-rich major satellite repeat (MSR) sequences that underlie the pericentric regions of each mouse chromosome [2–4]. These pericentric regions define constitutive heterochromatin and can comprise large arrays of > 10,000 copies of a reiterated 234 bp MSR unit [5]. Based on the A/T-richness and high copy number of MSR sequences, constitutive heterochromatin can be visualized by DAPI staining and is reflected by the classic clustering of heterochromatic foci. The integrity of this heterochromatin organization is crucial to secure accurate segregation of chromosomes [1], but is often altered in cancer cells that display perturbed heterochromatin organization and genomic instabilities [6, 7].

Heterochromatin is not transcriptionally inert [8, 9] and a certain fraction of the MSR units maintain transcriptional competence and generate major satellite repeat RNA [10]. RNA components have been suggested to stabilize heterochromatin formation [11] and to support binding of heterochromatin protein 1 (HP1) [12]. More recently, MSR RNA was shown to facilitate recruitment and retention of the core heterochromatin enzymes, the Suv39h H3K9 methyltransferases [13, 14]. While transcriptional activity of MSR DNA is mostly silenced in differentiated cells, MSR transcripts are up-regulated in the fertilized mouse zygote [15, 16], where they are required for heterochromatin formation and progression of early embryogenesis. Expression of some satellite transcripts has also been reported to increase upon heat shock [17] and to reflect a general stress response in human cells [18]. In addition to these physiological functions, regulation of satellite RNA expression is also relevant for pathological states. For example, satellite repeat transcripts are aberrantly elevated in several forms of human cancer [19] and forced over-expression of MSR transcripts in mouse mammary glands has been shown to be a tumor driver [20]. High levels of satellite repeat transcripts can induce transient RNA:DNA hybrid formation resulting in repeat element expansion and chromosome mis-segregation [21]. Despite these important insights, the regulation of satellite and, in particular of MSR transcriptional activity, is not fully understood.

The isoflavone genistein is a natural compound that is frequently used in dietary supplements to provide health benefits [22]. In addition, genistein has also been associated with reducing cancer risk [23–25]. Genistein has many described functions including it being a plant phytohormone and weak agonist for the estrogen receptor ERβ [26]. Genistein was also shown to be an inhibitor for tyrosine kinases [27] and to block DNA topoisomerase 2 [28]. Further, genistein has been described to activate the Nrf2 antioxidant response [29]. These diverse bioactivities of genistein appear to be dose-dependent and low concentrations (i.e. < 1 μM) of genistein agonize with the estrogen receptor pathway, but high concentrations (i.e. > 10–100 μM) inhibit cell growth [26]. Accordingly, health benefits of phytoestrogens and genistein in dietary supplements have been critically reviewed [22] and isoflavones and genistein can be both carcinogenic or cancer-protective [25]. The anticancer function of genistein has been associated with a high-dose treatment that inhibits tyrosine kinases and DNA topoisomerase 2 and which arrests cell cycle progression and induces apoptosis [30–34].

In this study, we identified the isoflavone genistein as a novel inducer for the transcription of MSR sequences in mouse heterochromatin. This genistein-mediated stimulation of MSR transcription is phenocopied by pharmacological poisoning of the DNA topoisomerases Top1 and Top2. These data provide new insight into the transcriptional response of mouse heterochromatin and suggest that transcriptional regulation of the MSR repeat units is guided by an altered topology of the underlying A/T-rich DNA sequence. Importantly, the data also reveal a novel function for genistein in stimulating MSR transcription that may contribute to the growth inhibitory and anticancer properties of this natural compound.

## Methods

### Tissue culture and cell lines

Mouse embryonic fibroblasts (MEF cells) [35] were cultured at 37** °C** and 5% CO_2_ in high glucose DMEM (Sigma Aldrich, D6171) supplemented with 10% FBS (Gibco, #10270), 2 mM L-Glutamine (Sigma Aldrich, G7513), 0.05 mM β-Mercaptoethanol (Sigma Aldrich, M7522), 1 mM Sodium-Pyruvate (Sigma Aldrich, S8636), and 1X non-essential amino-acids (Sigma Aldrich, M7145) [35].

For drug treatments, compounds were dissolved either in DMSO (Sigma Aldrich, D418) or saline solution (Sigma Aldrich S8776) and cells were treated with final concentrations and durations as indicated in the Appendix.

Heat shock was performed for 1 h in a 42 °C water bath, followed by a recovery for 3 h at 37 °C. Serum starvation was conducted for 24 h by utilizing high glucose DMEM medium (as above) but without FBS (0% FBS).

The C2C12 mouse myoblast cell line [36] was obtained from Andrew Pospisilik (then MPI-IE, Freiburg). Differentiation into C2C12 mouse myotubes was induced in high glucose DMEM medium (as above) containing 2% horse serum (Sigma Aldrich, H1138) instead of 10% FBS and the medium was replaced every second day.

*Top2β*-null MEF cells [37] were kindly gifted by C. Austin (University of Newcastle) and André Nussenzweig (NCI, Bethesda).

### Cell cycle and FACS analysis

1 × 10^5^ cells were plated in 10 cm dishes. After the drug treatments, cell pellets were collected and washed with cold PBS (Sigma Aldrich, D8537), centrifuged for 3 min at 500 g and resuspended in 70% cold ethanol under mild vortexing conditions. The cell suspension was then incubated overnight at 4 °C. The cell suspension was pelleted at 500 g and resuspended in 1 ml PBS containing 40 μg/ml Propidium Iodide (Sigma Aldrich, P4864) and 100 μg/ml RNase A (ThermoFisher, EN0531). Samples were subsequently incubated at 37 °C for 30 min in a water bath and transferred into FACS tubes through a 40 μm mesh-filter. Analysis of Propidium Iodide signal indicating DNA content was performed on a Fortessa I FACS sorter (BD Biosciences).

### RO-3306 cell synchronization

0.75 × 10^5^ cells were seeded onto 10 cm plates. The control samples were incubated for 24 h in media containing vehicle alone (DMSO) and washed twice with PBS before the addition of fresh media containing either DMSO alone or 50 μM genistein (dissolved in DMSO) for 24 h.

RO-3306 synchronization was performed by treating cells with 10 μM RO-3306 for 24 h. After, the plates were washed twice with PBS and then subjected to vehicle alone, 50 μM genistein or 50 μM genistein in combination with 10 μM RO-3306.

### Apoptotic indices

1 × 10^6^ cells were pelleted at 500 g and washed with PBS. For the detection of apoptotic indices, a BD Annexin V-FITC Apoptosis Detection Kit I (BD Biosciences, #556547) was used. Annexin V-FITC/Propidium Iodide signals, indicating pre-apoptotic/apoptotic cells were measured on a Fortessa I FACS sorter (BD Biosciences).

### Detection of LINE L1Md_A and MSR transcripts by directed RT-qPCR

1 × 10^5^ cells were plated in 10 cm dishes. After drug treatments, the cells were pelleted and lysed in 1 ml of Tri-Reagent (Sigma Aldrich, T9424). Instruction for RNA purification was followed except for substituting chloroform with 1-bromo-3-chloropropane (Sigma Aldrich, B62404). The purified total RNA was resuspended in nuclease-free water (Qiagen).

5 μg of total RNA was subjected to DNase digestion using TURBO DNase (ThermoFisher, AM2238). To ensure effective removal of MSR DNA, RNA was incubated for 1 h at 37 °C in a Thermomixer with 6 U of TURBO DNase. Thereafter, RNA was recovered using an RNA Clean & Concentrator-5 kit (Zymo Research, R1015). The TURBO DNase and RNA purification steps were repeated and the RNA was eluted in nuclease-free water (Qiagen). The integrity of DNase digested RNA was routinely analyzed using a 5300 Fragment Analyzer (Agilent).

1 μg of DNase digested total RNA was used for cDNA synthesis using the SuperScript II Kit (ThermoFisher, #18064014) with random hexamer primers (ThermoFisher, SO142). The cDNA was then diluted to a final concentration of 8 ng/μl with nuclease-free water (Qiagen). To assess for the presence of DNA contamination, RT-minus samples were prepared during cDNA synthesis. Amplification plots for PCR products in RT-plus and RT-minus samples were used to determine Ct-values and to verify differences by > 10 amplification cycles. This confirmed RT-minus samples to have negligible DNA.

16 ng of cDNA per reaction were pipetted in 384-well plates (ThermoFisher, BC3384) and amplified with a SybrSelect Master mix (ThermoFisher, #4472908), nuclease-free water and 250 nM final concentration of target-specific primer pairs in a total volume of 10 μl. The expression analysis was assessed on a QuantStudio 6 Flex qPCR system (Thermo Fisher) with an annealing temperature of 60 °C for 40 cycles.

Specific primers for the amplification of MSR, LINE L1Md_A 5’-UTR, Hprt or 5S rRNA are listed in the Appendix.

### HiSeq RNA sequencing

Total RNA was extracted and HiSeq RNA sequencing on ribosomal RNA depleted cDNA libraries was performed by the MPI-IE Deep Sequencing facility on either HiSeq2500 (Illumina) or NovaSeq6000 (Illumina) platforms. 150 bp paired-end reads (37.5 × 10^6^ reads/sample of either untreated and genistein-exposed MEF cells or 180 × 10^6^ reads/sample of wt and *Top2β*-null MEF cells) in fastq files were first trimmed (stringency 2) using Trim Galore! (v0.4.0) and aligned to the mm10 mouse genome using STAR (v2.5.2) [38] with the following parameters:

–outFilterMultimapNmax 100 –outAnchorMultimapNmax 100 –outSAMtype.

BAM Unsorted. The resulting alignment file was sorted by name using samtools v1.3.1 [39]. TEtranscripts v1.5.1 [40] was run with a modified mm10 repeatmasker GTF file, which was created by separately annotating the 5’UTR or ORF1 of LINE L1 elements, as previously described [14]. The annotations were converted to a GTF file using a custom rmsk2tetranscriptgtf.pl script. In addition, the Gencode M9 gene annotations were used with TEtranscripts to quantify coverage of the RNA-seq datasets over both genes and repeat elements.

Using the counts table generated from TEtranscript, differential expression analysis of both repeats and genes was performed using DESeq2 [41]. Data visualization was performed using ggplot2 [42]. Heatmaps were created using pheatmap in R (Kolde, 2015: Pretty Heatmaps. R package version 1.0.8. https://CRAN.R-project.org/package=pheatmap).

The results from differential expression analysis were filtered for minimum expression (baseMean > 100, log2FoldChange > 1 or < − 1) and significance (adjusted p-value < 0.05), and were used as inputs for further analysis. The canonical pathways were obtained through IPA (Qiagen Inc, 2014).

### De novo assembly of MSR contigs

Genomic DNA was isolated from mouse wt26 ES cells [43]. Nanopore long-read DNA sequencing (mean read length ≈ 13 kb), ultra-long DNA sequencing (mean read length ≈ 18 kb) and adaptive sequencing (mean read length ≈ 21 kb) was used for library preparation and processed by the MPI-IE Deep Sequencing facility. The initial processing of raw Nanopore sequencing data (fast5 format) was conducted using Guppy (Oxford Nanopore Technologies) configured to the high-accuracy mode for base-calling. Around 5,066 million of filtered reads were selected for the de novo assembly of MSR contigs. This de novo assembly was facilitated by flye v.2.9-b1768 algorithm that is using repeat graphs as a core data structure [44]. In total, 89 MSR scaffolds/contigs could be constructed that define pericentric MSR arrays at ten mouse chromosomes and together provide sequence information for > 18,018 MSR copies. The de novo assembly and full sequence information of these pericentric MSR contigs (GE_assembly4.0) will be detailed elsewhere.

### MSR RNA read alignment to the GE2(4821) and GE13(390) MSR contigs

GE2(4821) is a pericentric MSR contig of mouse chromosome 2 containing 4821 MSR copies and GE13(390) is a pericentric MSR contig of mouse chromosome 13 containing 390 MSR copies. HiSeq RNA sequencing reads were trimmed using cutadapt v.2.5 [45] and then aligned to the de novo assembly of MSR copies (GE_assembly4.0) with bwa mem v.0.7.17 [46]. The resulting BAM files were sorted using samtools v.1.9.0 [39] and bigwig files were generated through deeptools v.3.3.0 [47] with parameters “–samFlagExclude 384 –bs 5 –extendReads 5 –normalizeUsing RPGC –effectiveGenomeSize 2652783500”.

### Northern blot for MSR RNA

Northern blot analysis was performed using a NorthernMax-Gly Kit™ (ThermoFisher, AM1946). 10 μg of total RNA per sample was diluted 1:1 in a glyoxal/DMSO solution and denatured for 30 min at 55 °C. The RNA was separated on a 1% glyoxal/DMSO gel at 5 V/cm for 3 h by gel electrophoresis. Integrity and separation of RNA was assessed using an UV-transilluminator and the RNA was then transferred O/N onto a positively charged nylon membrane (GE Healthcare, RPN203S). Nucleic acids were crosslinked to the membrane by UV exposure at 365 nm for 3 min in a PeqLab Bio-Link BLX-365 UV-crosslinker. Blocking of the membrane was performed for 1 h using ULTRAhyb-Oligo buffer containing 25% formamide (ThermoFisher, AM8663) at 48 °C in a hybridization oven.

A strand specific 44nt probe (MSR-SR4) (see Appendix) complementary to the forward strand of major satellite transcripts was end-labelled with ATP[γ-^32^P] (Perkin Elmer, NEG502A250UC) using T4 PNK (NEB, m0201). Probe activity was measured using a scintillation counter (Perkin Elmer Tri-Carb 2910 TR) and the probe was added directly to the blocking/hybridization buffer to a final concentration of 10^6^ cpm/ml. Hybridization was performed overnight at 48 °C rotating in a hybridization oven, washed twice for 30 min at 48 °C with wash buffer (2X SSC and 0.5% SDS). A phosphorscreen was exposed to the membrane and scanned on a Typhoon FLA 9500 laser scanner (GE Healthcare).

### RNA-FISH analysis for MSR RNA

2 × 10^4^ cells were seeded on 4-well chamber glass slides (Corning, 154534PK) and left untreated (DMSO alone) or exposed to 50 μM genistein, either with or without 1 μM JQ1. 24 h after treatment, cells were briefly washed with cold PBS and subsequently washed with CSK buffer (100 mM NaCl, 3 mM MgCl_2_, 300 mM sucrose, and 10 mM PIPES pH 6.8) for 30 min. The cells were then permeabilized in CSK buffer supplemented with 0.5% Triton X-100 and 10 mM ribonucleoside vanadyl complex (RVC) (NEB, S1402S) for 3 min on ice. After permeabilization, the cells were washed with CSK with 10 mM RVC for 30 s on ice and then quickly fixed in 4% paraformaldehyde (ThermoFisher, 28,908) for 10 min at RT. To prepare the RNase A control, the slides were rinsed with PBS for 5 min at RT and then treated with 0.5 mg/ml RNase A (ThermoFisher, EN0531) in PBS for 20 min at RT. Slides were then washed in PBS for 5 min at RT. The slides were then rinsed in ice-cold 70% ethanol and subjected to a dehydration series. Slides were placed in increasing concentrations of ice-cold ethanol (70%, 80%, 90%, 100%) for 3 min at each step. After the final 100% ethanol dehydration step, the slides were allowed to air dry at RT. As the slides were air drying, the biotinylated locked nucleic acid (LNA) probes against MSR RNA (see Appendix) were prepared for hybridization. Four probes, detecting either the sense or antisense transcripts were diluted to a final concentration of 200 nM for each probe in 30% formamide in water and denatured at 80 °C for 10 min and then immediately placed on ice for 10 min. An equal volume of hybridization buffer (0.4% BSA, 4X SSC, 20% dextran sulfate, and 40 mM RVC) was then added. Probes were used at a final concentration of 100 nM of each of the four probes. This hybridization mixture was then placed on the slides and incubated in a humid chamber at 37 °C for 30 min. After hybridization, slides were then washed in a series of increasing stringent buffers: 1) 15% formamide in 2X SSC for 20 min at 37 °C, 2) 0.1X SSC for 20 min at 60 °C, 3) 1X SSC for 20 min at RT, and 4) 4X SSC for 2 min at RT. To detect the probes, streptavidin-Cy3 (Sigma Aldrich) was diluted 1:5000 in detection buffer (4X SSC, 1% BSA, and 40 mM RVC) and incubated on the slides for 1 h at RT. The slides were subsequently washed with the following buffers for 10 min at RT: (1) 4X SSC, (2) 4X SSC, 0.1% Triton X-100, and (3) 4X SSC. The slides were then mounted using VectaShield™ containing DAPI and analyzed on an Apotome 2 (Zeiss) microscope. MSR RNA FISH puncta per nucleus were counted manually.

### Indirect immunofluorescence

1 × 10^4^ cells were seeded on 8-well chamber glass slides (Corning, #154461PK) the day before processing. Cells were fixed with 4% paraformaldehyde (Sigma Aldrich, #1004968350) for 10 min at RT and subsequently washed 3 × 5 min in PBS. Permeabilization was performed with 0.5% Triton X-100 in PBS for 5 min at RT, followed by 3 × 5 min PBS washes. Slides were incubated with primary antibodies against α-53bp1 (Abcam ab36832, 1:200), α-γH2A.X (Milipore 05–636, 1:500) or α-Rpa2 (Cell signaling 2208, 1:200), diluted in PBS supplemented with 5% goat serum for 1 h. Slides were washed 3 × 5 min in PBS, and then secondary antibodies conjugated to Cy3/Cy5 (goat α-mouse Cy3, A10521 Invitrogen; goat α-rabbit Cy5, A10523 Invitrogen; goat α-rat Cy3, A10522 Invitrogen; all 1:250 dilution) were added for 1 h at RT in PBS supplemented with 5% goat serum. Slides were then washed 3 × 5 min with PBS, mounted using VectaShield™ containing DAPI (Vectorlabs, H-1200) and sealed with nail polish.

Fluorescence signals were acquired on an Apotome 2 (Zeiss) confocal microscope using an EC-Plan Neofluar NA 1.30 40 × oil objective with Immersol™ immersion oil (Zeiss). Zen 2 (Zeiss) imaging software was used to collect images.

### MNase chromatin accessibility assay

4 × 10^5^ were plated on 15 cm dishes. The cells were left untreated (DMSO alone) or exposed to 50 μM genistein and 24 h later a cell pellet was harvested. The cell pellet was resuspended to a final concentration of 1–2.5 × 10^7^ cells per ml in ice-cold hypotonic buffer (20 mM HEPES pH 7.5, 20 mM NaCl, 5 mM MgCl_2_, and 0.1% NP-40) with an 18G needle and syringe and left on ice for 10 min. The cell mixture was then centrifuged at 500 g for 5 min at 4 °C. The supernatant was removed and the nuclei were washed two times with Ex100 (20 mM HEPES pH 7.5, 100 mM NaCl, and 0.5 mM MgCl_2_). After the last wash, the nuclei were resuspended in Ex100 and kept on ice for 15 min. The nuclei were centrifuged at 500 g for 5 min at 4 °C. The pellet was resuspended in 1 ml of Ex100 and the concentration of nucleic acids was estimated using a NanoDrop 1000 (ThermoFisher). The nuclei were then diluted to a final concentration of 500 ng/μl nucleic acids. 1 ml of suspended nuclei was then incubated with CaCl_2_ to a final concentration of 2 mM for 10 min at 25 °C. 100 μl of sample was aliquoted into five tubes and incubated with 0, 3, 6, 9, and 12 U of MNase (ThermoFisher, EN0181) for 10 min in a heating block set to 25 °C. The reaction was stopped with the addition of EDTA to a final concentration of 10 mM. The DNA was then purified using a PCR purification kit (Jena Bioscience, PP-201L). The resulting DNA was electrophoresed on an 1% agarose gel to visualize the digestion pattern of the DNA.

### Western blotting

1 × 10^5^ cells were plated in 10 cm dishes. After drug treatments, cells were pelleted at 500 g for 5 min, washed once in cold PBS and lysed in RIPA buffer (ThermoFisher, #89900) supplemented with protease (Sigma Aldrich, #11836170,001) and phosphatase inhibitors (Sigma Aldrich, PHOSS-RO). Samples were then incubated on ice for 5 × 10 min with mild vortexing every 10 min. The cell lysate was then sonicated using a Bioruptor (Diagenode) and protein concentration was measured with a Bradford assay using the Bio-Rad Protein Assay Dye Reagent Concentrate (Biorad, #5000006). 4X protein loading dye with a final concentration of 0.1 M DTT and water was added to 10 μg of protein sample per well, boiled at 95 °C for 5 min, loaded on a 4–20% SDS-PAGE gradient gel (BioRad, #4561096) and separated at 200 V for 30 min using a BioRad mini-PROTEAN electrophoresis system.

Wet transfer was performed on PVDF membrane (Merck, IPVH00010) at 100 V for 1 h. Membranes were blocked using a 5% w/v milk powder in TBS-T (0.1% tween-20) for 1 h rotating at RT. The following primary antibodies were diluted in fresh blocking solution: α-γH2A.X (Milipore 05–636, 1:1000), α-H3 (Abcam ab1926, 1:10,000), α-Gapdh (Abcam ab8245, 1:10,000), α-Tubulin (Abcam ab4074, 1:1000) and incubated rotating O/N at 4 °C. Membranes were then washed 3 × 10 min in TBS-T and incubated with secondary antibodies conjugated to HRP (goat α-rabbit HRP, 111–035-133 Jackson Labs; rabbit α-mouse HRP, 315–035-006 Jackson Labs; all 1:2500 dilution) in fresh TBS-T with 5% w/v milk powder for 1 h. After 3 × 10 min washes with TBS-T, membranes were soaked in ECL solution (ThermoFisher, #32106) for 5 min and the signal was detected using Amersham ECL Hyperfilm (GE, #28906837).

### siRNA knockdown of Top1, Top2α and Top2β

siRNA was purchased from DHARMACON (Top1: L-047567-01-0005; Top2α: L-043916-01-0005; Top2β: L-042134-00-0005) and transfected at 37.5 nM final concentration in 10 cm dishes containing 1 × 10^5^ cells (seeded a day before transfection) using the transfection reagent DharmaFect I (DHARMACON, T-2001). Cells were incubated with siRNA for 48 h and RNA and protein was extracted.

## Results

### Genistein massively stimulates major satellite repeat expression in mouse fibroblast cells

**Fig. 1:**
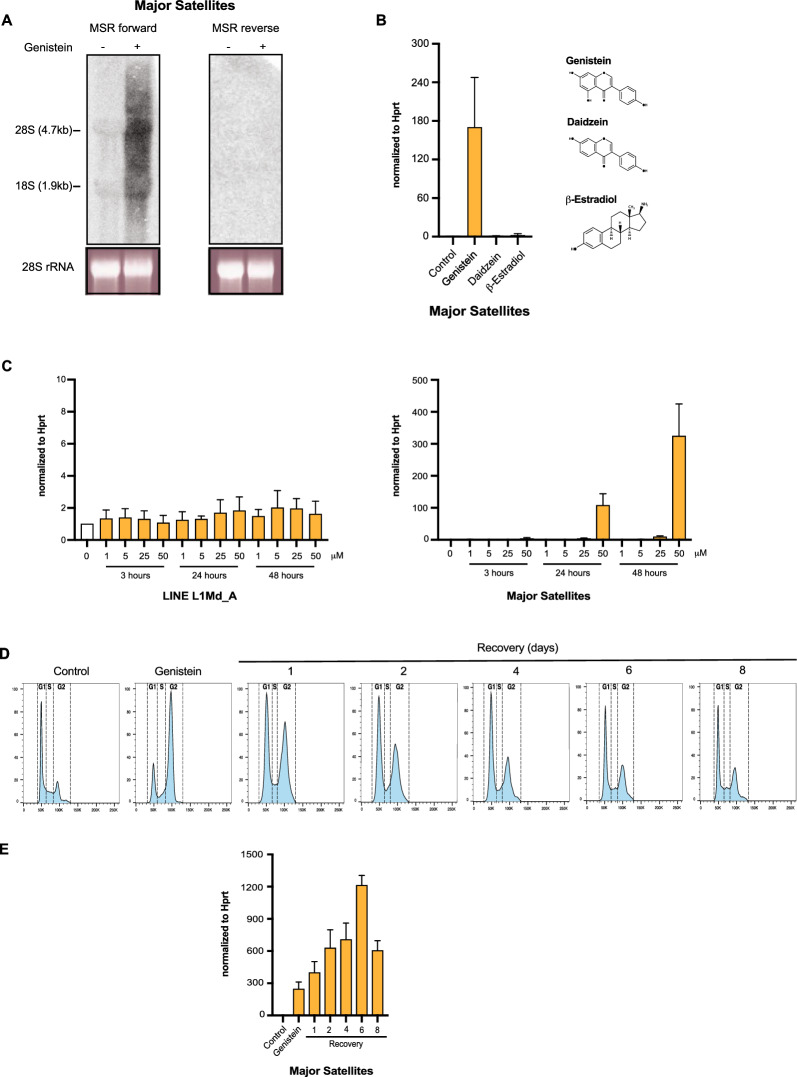
Genistein massively stimulates major satellite repeat expression in MEF cells. **A** Northern blot of total RNA from untreated and genistein-exposed (24 h) MEF cells with strand-specific MSR probes detecting forward or reverse major satellite repeat transcripts. Integrity of RNA is verified by staining of 28S rRNA. **B** RT-qPCR for MSR transcripts after a 24 h incubation of MEF cells with genistein, daidzein or β-estradiol. Values are normalized to *Hprt* and are relative to the control (DMSO alone). Error bars reflect the standard deviation from n = 3 biological replicates. **C** RT-qPCR for LINE L1Md_A (left panel) and MSR (right panel) transcripts in MEF cells exposed to increasing incubation times (3 h, 24 h, 48 h) and increasing concentrations (1 to 50 μM) of genistein. Values are normalized to *Hprt* and are relative to the control (DMSO alone). Error bars reflect the standard deviation from n = 3 biological replicates. **D** FACS profiles for cell cycle stages in MEF cells at 1, 2, 4, 6 and 8 days after wash out of genistein. **E** RT-qPCR for MSR transcripts in MEF cells at 1, 2, 4, 6 and 8 days after wash out of genistein. Values are normalized to *Hprt* and are relative to the control (DMSO alone). Error bars reflect the standard deviation from n = 3 biological replicates

This study was stimulated by the notion that several components of mammalian heterochromatin participate in signaling pathways [48] and can engage in stress response [49]. Further, heat shock induced accumulation of heterochromatic satellite repeat transcripts has been proposed to be a general stress response in human cells [18]. We examined stress response and several signaling pathways, such as JNK and PI3 kinases, TGFβ and antioxidant signaling by using small molecule compounds (data not shown). Among the compounds that we tested, we identified the isoflavone genistein as a major inducer for MSR transcription. We incubated mouse embryonic fibroblasts (MEF cells) with increasing concentrations and for different exposure times with genistein and then analyzed MSR transcripts by Northern blot. A 24 h incubation with 50 μM genistein significantly augmented MSR forward (purine-rich) transcripts but did not induce MSR reverse (pyrimidine-rich) transcripts (Fig. 1A). MSR RNA is detected in a broad size range (from < 500 bp to > 8000 bp) that reflects a heterogenous population of MSR transcript as described in previous reports [9]. We then quantified this MSR transcriptional stimulation by RT-qPCR with MSR specific primers (Fig. 1B). This RT-qPCR is a cluster analysis that detects all MSR transcripts that are identical to the MSR consensus sequence (supplementary Figure S1), but it cannot discriminate distinct MSR variants within the pericentric arrays or at intergenic locations. Cells start to respond to concentrations of 50 μM genistein in a 24 h exposure with an around 100-fold accumulation of MSR transcripts that is even more pronounced after 48 h. By contrast, transcripts for other repeat classes, such as the LINE L1Md_A elements are not elevated (Fig. 1C). A genistein derivative (daidzein) lacking a hydroxyl group in the isoflavone backbone or the hormone β-estradiol did not induce accumulation of MSR transcripts (Fig. 1B). FACS analyses for cell cycle profiles indicated that a 24 h treatment with 50 μM genistein results in a major transition of the cell cycle, such that most cells are shifted to the G2/M phase. Removing genistein after 24 h and cultivating the cells in normal medium shows that genistein-stressed MEF cells need 6–8 days to recover their normal cell cycle profile (Fig. 1D). During this recovery phase, MSR transcripts increasingly accumulate to more than 1000-fold (day 6 of recovery) until they start to decline, however still with very high levels of MSR transcripts (Fig. 1E). The genistein-mediated accumulation of MSR transcripts is much more pronounced as compared to the < 5-fold desilencing of MSR transcripts in *Suv39h* double-null MEF [35]. For all the subsequent analyses on the genistein-mediated stimulation of MSR transcription, we used a 24 h exposure to 50 μM genistein, which on average, induces an 80- to 120-fold accumulation of MSR transcripts.

### Selective accumulation of major satellite repeat transcripts following genistein exposure

**Fig. 2:**
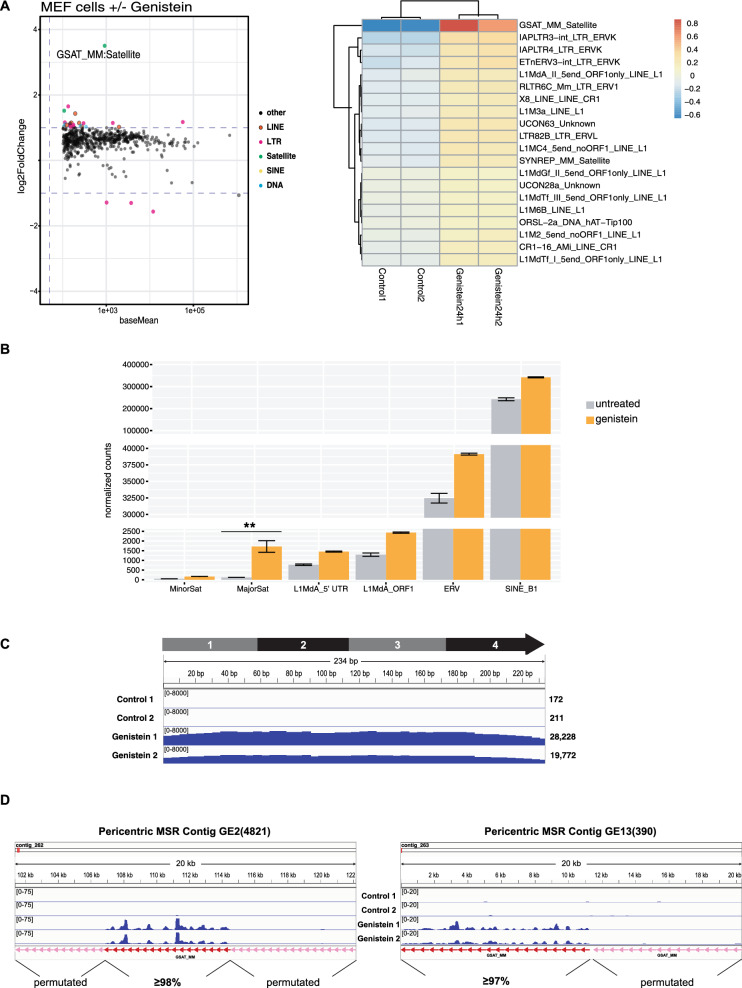
HiSeq RNA sequencing and meta analysis of repeat element expression in untreated and genistein-exposed MEF cells. **A** MA plot and heat map for repeat element expression (LINE, LTR-ERV, SINE and satellite repeat classes) in HiSeq RNA libraries from untreated and genistein-exposed MEF cells (n = 2). Dashed lines in the MA plot discriminate non-significant expression differences that are below the cut-off (baseMean > 100, log2FoldChange > 1 or < -1). GSAT_MM denotes major satellite repeats. **B** Quantification of normalized read counts for minor satellite, major satellite, LINE L1Md_A, ERV and SINE repeat classes. Asterisks indicate statistically significant differences above a twofold threshold when compared to untreated control (**p < 0.01, FDR/Benjamini-Hochberg, n = 2). **C** Cumulative alignment of clustered MSR reads in untreated and genistein-exposed MEF cells to one unit of the MSR consensus DNA sequence (234 bp) (n = 2). **D** Alignment of MSR reads to intact (> 97% identity to MSR consensus, indicated by red arrows) or permutated MSR copies (indicated by pink arrows) in two examples (GE2(4821) and GE13(390)) of de novo assembled pericentric MSR contigs. For each example, a 20 kb segment of reiterated MSR units (indicated by red and pink arrows, but not to scale) is shown

We next performed HiSeq total RNA sequencing in untreated and genistein-treated MEF cells to identify the response profile of all repeat classes in the mouse genome. The MA plot and heatmap shows that MSR RNA sequences (GSAT_MM_Satellite) are the most significantly up-regulated and that the other repeat classes, such as endogenous retroviruses (ERV) and LINE elements only display a modest increase (Fig. 2A). Expression of the distinct repeat classes was then quantified by using the sum of normalized read counts within each repeat class. In untreated cells, SINE elements are most highly expressed (around 200,000 reads), followed by ERV (around 30,000 reads) and LINE repeats (around 1000 reads). Major satellite repeat reads and minor satellite repeat reads are only detected at very low numbers above the base cut-off (minimal read cut-off = 100 reads) (Fig. 2B). Genistein treatment particularly elevated MSR transcripts to around 1,500 reads, but resulted in only a minor increase for SINE, ERV and LINE reads. Also, minor satellite repeat reads were not significantly up-regulated. We conclude from these data that genistein exposure of MEF cells selectively induces up-regulation of MSR transcripts.

We also compared cumulative (multi-mapping) MSR transcripts and aligned them to one unit of the MSR consensus sequence (Fig. 2C). While untreated MEF cells have only very low MSR transcript levels (around 200 accumulated reads), this number increases to > 20,000 accumulated reads after genistein exposure (Fig. 2C). This magnitude of MSR transcript up-regulation is similar to the more than 100-fold accumulation that we detect by RT-qPCR. We note, however, that RT-qPCR is likely to overestimate the quantification of MSR transcripts since there will be several target sites for PCR primers in heterogenous MSR cDNA transcribed through multiple MSR units. Read-level quantification by HiSeq total RNA sequencing indicated a < 15-fold increase for MSR transcripts reads following genistein exposure (Fig. 2C). Since comparative expression analyses in most of the subsequent experiments are done by RT-qPCR (and not by HiSeq RNA sequencing), we refer to RT-qPCR approximation for relative MSR transcript abundance.

We next used long-read DNA sequencing to allow for a de novo assembly of pericentric DNA contigs containing reiterated copies of MSR units (see Methods). With this bioinformatic de novo assembly, we obtained sequence information for > 18,000 MSR copies (data not shown). The pericentric MSR units are organized in head-to-tail tandem arrangements and comprise of MSR copies with high sequence identity (> 97%) to the MSR consensus sequence [5] but also many MSR variants that are considerably permutated. We chose two examples of these pericentric MSR contigs, GE2(4821) on mouse chromosome 2 and GE13(390) on mouse chromosome 13, to illustrate genistein-mediated transcriptional response of MSR units (Fig. 2D). Following genistein exposure, we detect up-regulation of MSR transcripts and can align some of these MSR transcript reads to the GE2(4821) and GE13(390) pericentric MSR contigs. Intriguingly, MSR transcript reads cluster with MSR units that have high sequence identity (> 97%) to the MSR consensus sequence and we cannot identify MSR transcript reads that would align to permutated MSR variants (Fig. 2D). These data are consistent with a model that only intact MSR units with a full number of described transcription factor binding sites (see supplementary Figure S1) maintain transcriptional competence and initiate MSR transcripts [10]. While these alignments were derived from 150 bp paired-end RNA reads, we are not excluding that long-read RNA sequencing could detect heterogenous MSR RNA that also contains read-through transcripts into permutated MSR variants.

### Genistein-mediated accumulation of MSR transcripts requires active transcription by RNA polymerase II

**Fig. 3:**
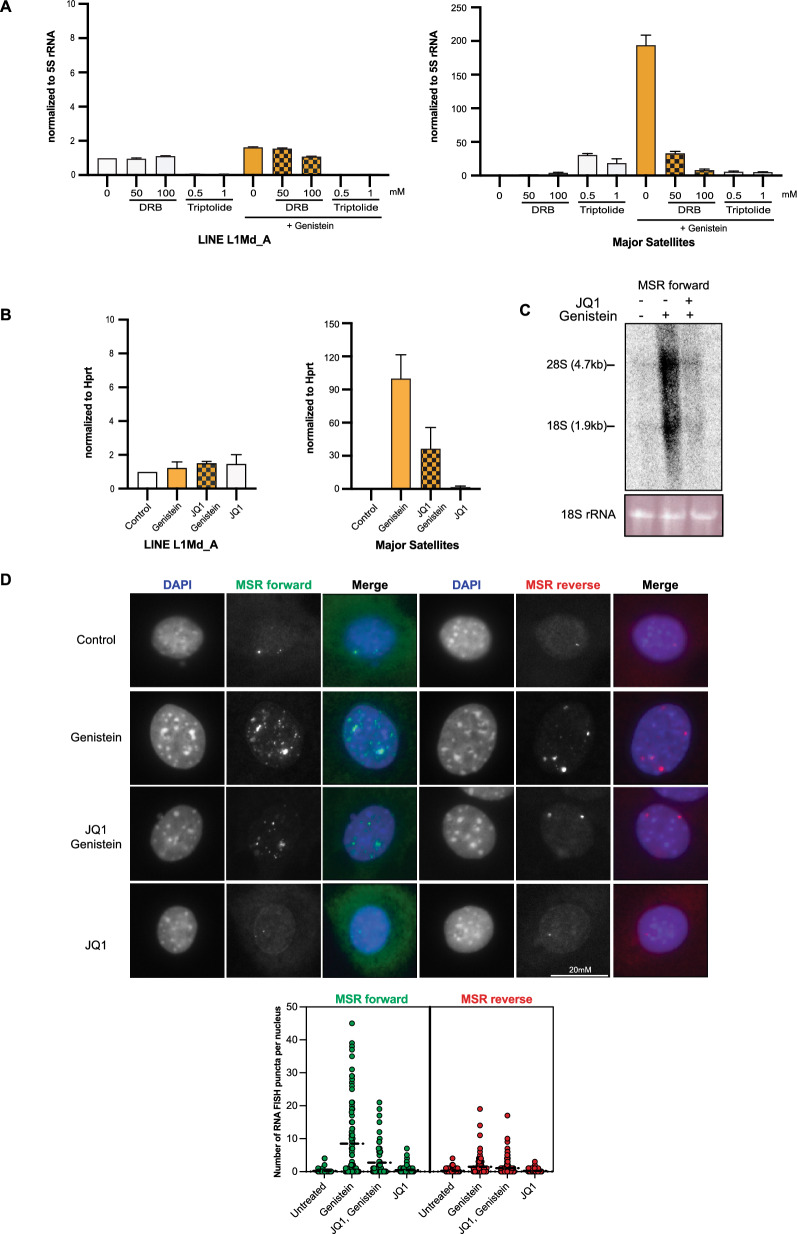
Genistein-mediated accumulation of MSR transcripts requires active transcription by RNA polymerase II. **A** RT-qPCR for LINE L1Md_A (left panel) and MSR (right panel) transcripts from untreated and genistein-exposed MEF cells that were incubated with the RNA pol II inhibitors DRB and triptolide. Values are normalized to 5S rRNA, which is an RNA pol III transcript not inhibited by DRB. Error bars reflect the standard deviation from n = 3 biological replicates. **B** RT-qPCR for LINE L1Md_A (left panel) and MSR (right panel) transcripts from untreated and genistein-exposed MEF cells that were also incubated with the BRD4 inhibitor JQ1. Values are normalized to *Hprt* and error bars reflect the standard deviation from n = 3 biological replicates. **C** Northern blot of total RNA from untreated, genistein-exposed and genistein/JQ1 treated MEF cells with a strand-specific MSR probe detecting forward major satellite transcripts. Integrity of RNA is verified by staining of 18S rRNA. D) RNA FISH in untreated, genistein-exposed, genistein/JQ1 or JQ1 treated MEF cells with strand-specific MSR probes detecting forward or reverse major satellite repeat transcripts. Nuclei were counterstained with DAPI. For each sample, n ≥ 80 cells were analyzed. Scale bar is 20 μm. In the lower panel, violin plots quantifying the number of MSR RNA FISH puncta per nucleus are shown (green is forward transcript, red is reverse transcript)

The genistein-induced stimulation of MSR expression is not a fast transcriptional response and is not detected within 3 h of genistein exposure (Fig. 1C). We addressed whether genistein-mediated accumulation of MSR transcripts requires active transcription by RNA polymerases. We used 5,6-dichlorobenzimidazole 1-β-D-ribofuranoside (DRB) as a selective RNA polymerase II (RNAPII) inhibitor and triptolide as an even more global inhibitor of all RNA polymerase activities [50]. DRB significantly reduced the genistein-mediated stimulation of MSR transcription by around tenfold and triptolide nearly eliminated the detection of genistein-induced MSR transcripts. These data indicate RNAPII to be the key RNA polymerase for the genistein-induced accumulation of MSR transcripts. By contrast, LINE L1Md_A transcripts are not considerably inhibited by DRB treatment (Fig. 3A, left panel). Although we could not detect RNAPII enrichment at MSR chromatin either in untreated or genistein-exposed MEF cells (data not shown), these results indicate RNAPII to be the key RNA polymerase for the genistein-induced accumulation of MSR transcripts.

We further analyzed a direct involvement of ongoing transcription by using the JQ1 inhibitor, which blocks Brd4-assisted transcriptional stimulation [51]. JQ1 inhibition has recently been shown to attenuate stress response in human heterochromatin [52]. JQ1 is effective in reducing genistein-mediated up-regulation of MSR transcripts, both as measured by RT-qPCR (Fig. 3B) and by Northern blot (Fig. 3C). In addition, we also performed RNA-FISH that show considerably increased signals for the forward (purine-rich) and, to a slightly lesser degree, reverse (pyrimidine-rich) MSR transcripts after genistein exposure. Most of these increased MSR RNA-FISH signals overlap with or are in the periphery of DAPI-dense foci (Fig. 3D). Simultaneous JQ1 treatment of genistein exposed MEF cells reduces these up-regulated MSR transcripts and the corresponding number of MSR RNA FISH puncta per nucleus (Fig. 3D, bottom panel).

In genistein-stressed MEF cells, we have not observed dispersion of HP1α from heterochromatic foci or reduced heterochromatic H3K9me3 or H4K20me3 signals (supplementary Figure S2A). ChIP-qPCR analyses also indicated no significant change for H3K9me3 and HP1α at MSR chromatin in untreated or genistein-stressed MEF cells (supplementary Figure S2B). We also did not detect altered localization for Suv39h1 and Suv39h2 H3K9 KMT enzymes (supplementary Figure S2C) or for the high mobility group proteins Hmga1 and Hmga2 [48] (supplementary Figure S2D). We conclude from these analyses that genistein-mediated up-regulation of MSR transcripts requires active transcription by RNAPII but occurs without apparent changes in heterochromatin organization and in the localization of core heterochromatin components.

### Genistein activates stress signaling pathways and induces DNA damage

Ingenuity pathway analysis (IPA) analysis of the HiSeq RNA sequencing data for an altered gene response indicated that genistein activates key stress signaling and DNA damage pathways (e.g. up-regulation of AMPK and oxidative stress response and of p53 signaling) (supplementary Figure S3A). Upstream nodes for these stress responses are JNK and p38 MAPK signaling pathways [53]. However, genistein-mediated up-regulation of MSR expression was not significantly altered in *Jnk* double-null MEF cells or in *p53* null MEF cells (supplementary Figure S3B).

**Fig. 4:**
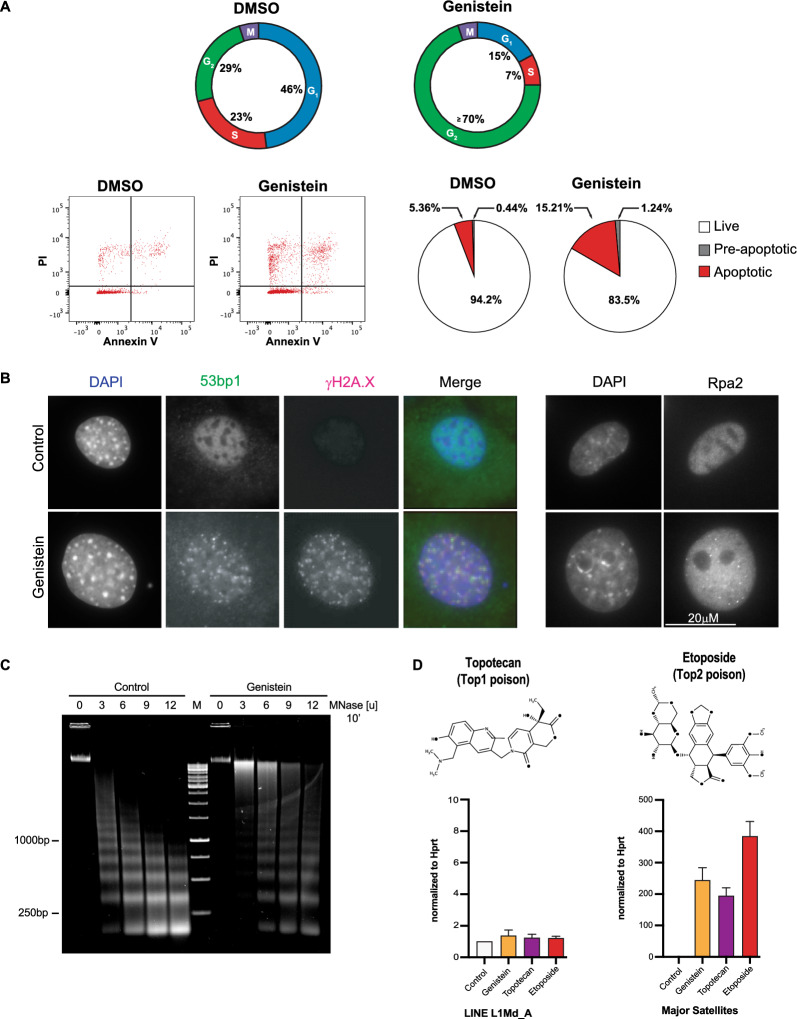
DNA damage and chromatin alterations induced by genistein exposure. **A** Cell cycle profiles and apoptotic indices of untreated (DMSO alone) and genistein-exposed MEF cells. A scheme representing the percentages of cells in distinct cycle stages (G1, S, G2) during asynchronous culture of the cell populations is summarized on top. FACS profiles (propidium iodide and Annexin V staining) (shown in left panel) were used to determine pre-apoptotic and apoptotic indices that are displayed in pie charts (right panel). **B** Immunofluorescence for the detection of 53bp1 and γH2A.X (left panel) or Rpa2 (right panel) in untreated or genistein-exposed MEF cells. Nuclei were counterstained with DAPI. For each sample, n ≥ 103 cells were analyzed. Scale bar is 20 μm. **C** Gel electrophoresis of genomic DNA fragmented from chromatin of untreated or genistein-exposed MEF cells with increasing units of MNase. DNA is stained with SYBR Safe. **D** RT-qPCR for LINE L1Md_A (left panel) and MSR (right panel) transcripts from MEF cells exposed for 24 h to genistein, a Top1 (Topotecan) or Top2 (Etoposide) poison. Values are normalized to *Hprt*. Error bars reflect the standard deviation from n = 3 biological replicates

Genistein treated cells have a larger nuclear size, show a shift in the cell cycle to the G2/M phase and increase a pre-apoptotic and apoptotic cell population (Fig. 4A). DNA damage after genistein exposure was analyzed by immunofluorescence (IF) for 53bp1 and γH2A.X. We also included IF for replication protein Rpa2, since this can detect single-stranded DNA and is an indicator for replicative stress. In genistein-treated MEF cells, there are pronounced and apparently overlapping signals for 53bp1 and γH2A.X. (Fig. 4B, left panels). Rpa2 signals are also broadly increased and display distinct Rpa2 puncta (Fig. 4B, right panel). Only very few of these DNA damage puncta coincide with DAPI-dense heterochromatic loci. In addition to DNA damage, genistein reduces the overall (bulk) accessibility of chromatin, as longer incubation times for nuclease fragmentation (MNase) of chromatin are needed (Fig. 4C).

Less accessible chromatin has been documented in UV damaged cells [54, 55]. DNA damage also occurs in cells that have blocked DNA topoisomerases [56] and one of the described functions for genistein is as a DNA topoisomerase 2 (Top2) inhibitor [28, 31, 33]. We therefore used a 24 h exposure of MEF cells to 1 μM etoposide (Top2 poison) and also to 1 μM topotecan (synthetic analog of camptothecin and Top1 poison) and examined accumulation of MSR transcripts by RT-qPCR. Intriguingly, both etoposide and topotecan phenocopy the genistein-mediated stimulation of MSR transcription, where etoposide induces an even greater  accumulation of MSR transcripts (350-fold) (Fig. 4D).

### G2/M blocked cells or post-mitotic cells show attenuated stimulation of MSR transcription following genistein exposure

**Fig. 5:**
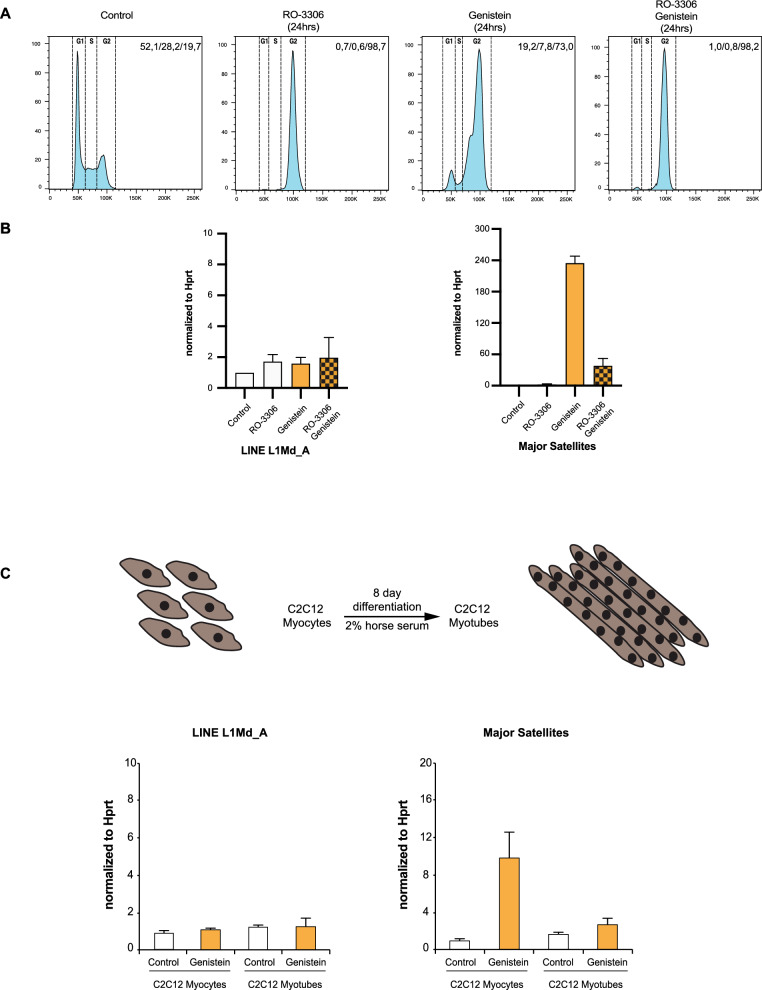
G2/M blocked MEF cells or post-mitotic C2C12 cells show attenuated stimulation of MSR transcription following genistein exposure. **A** FACS profiles for cell cycle stages in untreated, RO-3306 blocked, genistein-exposed and genistein-exposed/RO-3306 blocked MEF cells. Percentages for cell cycle stages (G1/S/G2) are indicated in the upper right corner of each FACS profile. **B** RT-qPCR for LINE L1Md_A (left panel) and MSR (right panel) transcripts from untreated, RO-3306 blocked, genistein-exposed and genistein-exposed/RO-3306 blocked MEF cells. Values are normalized to *Hprt*. Error bars reflect the standard deviation from n = 3 biological replicates. **C** RT-qPCR for LINE L1Md_A (left panel) and MSR (right panel) transcripts from untreated and genistein-exposed C2C12 myocytes and post-mitotic C2C12 myotubes. Values are normalized to *Hprt*. Error bars reflect the standard deviation from n = 3 biological replicates. A scheme for the 8 day cell culture differentiation of C2C12 myocytes into post-mitotic C2C12 myotubes is shown on top

The MEF cells used in this study have an average cell duplication time of around 12.5 h with 48–52% of the cells to be in the G1 phase of the cell cycle (Fig. 5A). A 24 h incubation with genistein induces a pronounced change in the cell cycle, such that most of the cells are shifted to the G2/M phase; however a reduced G1 population (< 19%) persists (Fig. 5A). We used a CDK1 inhibitor (RO-3306) to block MEF cells at the G2/M boundary and asked whether RO-3306 arrest would alter genistein-mediated stimulation of MSR transcription. Whereas a 24 h RO-3306 treatment alone did not result in the accumulation of MSR transcripts, a concurrent 24 h incubation with RO-3306 of genistein-stressed MEF cells significantly reduced genistein-mediated accumulation of MSR transcripts (Fig. 5B). In this experimental set-up, RO-3306 blocked and genistein stressed MEF cells largely lack a G1 cell population (< 1%) (Fig. 5A). These FACS profiles suggest that genistein-mediated stimulation of MSR transcription requires a considerable G1 cell population that is committed to progress into the next cell cycle.

These data are reminiscent of the proliferation dependent and cell cycle regulated transcription of MSR DNA [9], where it was also shown that MSR transcripts are largely absent in quiescent cells. To directly examine whether genistein-mediated stimulation of MSR transcription requires actively cycling cells or whether it is attenuated in non-cycling cells, we used mouse C2C12 myocyte differentiation into post-mitotic myotubes [36] by an 8 day cultivation in 2% horse serum. In cycling C2C12 myocytes, genistein increased MSR transcript levels by around tenfold, but there was no induction of MSR transcripts in C2C12 myotubes (Fig. 5C).

### Destabilizing and/or damaging chromatin structure can trigger MSR up-regulation

**Fig. 6:**
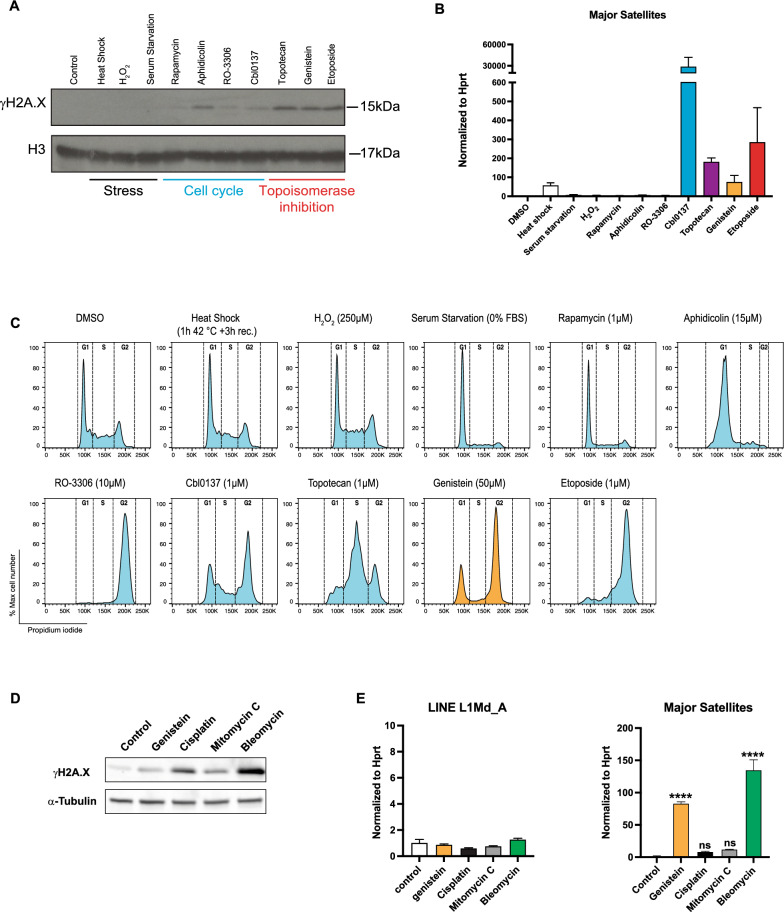
Chromatin and DNA damage can trigger MSR up-regulation. **A** Western blot for γH2A.X detection in MEF cells exposed to various stress signals (heat shock, H_2_O_2_, serum starvation), cell cycle blocks (rapamycin, aphidicolin, RO-3306), chromatin damage (Cbl0137) and topoisomerase poisoning (topotecan, genistein, etoposide). Histone H3 expression is used as a loading control. **B** RT-qPCR for MSR transcripts in MEF cells exposed to the conditions explained in **A**. Values are normalized to *Hprt*. Error bars reflect the standard deviation from n = 3 biological replicates. **C** FACS profiles (propidium iodide) for cell cycle stages in MEF cells exposed to the conditions explained in **A**. With the exception of the idealized FACS profile for genistein-exposed MEF cells (indicated in orange), all other FACS profiles were run in one experimental set up. **D** Western blot for γH2A.X detection in MEF cells exposed to genistein or the anticancer agents cisplatin, mitomycin C and bleomycin. Tubulin expression is used as a loading control. **E** RT-qPCR for MSR transcripts in MEF cells exposed to genistein or cisplatin, mitomycin C and bleomycin. Values are normalized to *Hprt*. Error bars reflect the standard deviation from n = 3 biological replicates. Asterisks indicate statistically significant differences (****p < 0.0001, ns is not significant, one-way ANOVA)

Genistein shifts the cell cycle and causes DNA damage, and Top1 and Top2 inhibitors or poisons phenocopy the genistein-mediated stimulation of MSR transcription. In order to start uncoupling these distinct processes and examine their possible contribution for MSR up-regulation, we compared various conditions that either do or do not induce DNA or chromatin damage. We used physiological stress, such as heat shock, peroxide and serum starvation and also blocked cell cycle progression (e.g. G1 or G2/M) with either rapamycin, aphidicolin and RO-3066. Moreover, we included a ‘chromatin-damaging’ agent (curaxin Cbl0137) that evicts histones and destabilizes nucleosomes but would only induce minimal DNA lesions [57]. Topotecan, genistein and etoposide were used to block topoisomerases resulting in the concomitant occurrence of DNA damage. We examined the response of MEF cells to each of these treatments by Western blot for γH2A.X (DNA damage) (Fig. 6A), FACS profile (cell cycle progression) (Fig. 6C) and Annexin V staining (apoptosis) (supplementary Figure S4). Although genistein also causes DNA damage, it has a much lower cytotoxic and apoptotic index as compared to topotecan or etoposide (supplementary Figure S4).

We then analyzed expression of MSR transcripts by directed RT-qPCR (Fig. 6B). The data indicate that we can, for the most part, identify two subgroups of stress conditions that significantly differ in the magnitude of MSR up-regulation. In the first subgroup, MSR transcription is augmented between 10- to 30-fold following heat shock and only marginally elevated with peroxide or serum starvation (Fig. 6B). No MSR up-regulation was detected with rapamycin (mTOR kinase inhibitor) and aphidicolin (DNA polymerase α inhibitor) (Fig. 6B) that both increase a G1 cell population or with RO-3306 which blocks a G2/M cell population. With the exception of aphidicolin, these conditions largely do not induce DNA damage (Fig. 6A). In the second subgroup, MSR transcription is massively augmented by between 100- 300-fold using either topotecan, genistein or etoposide (Fig. 6B). These conditions induce topoisomerase associated double-strand breaks (DSB) [58] and although they generate a high proportion of cells to be in the G2/M phase, they still maintain a cycling G1 cell subpopulation (Fig. 6C). Exceptionally, Cbl0137 treatment provoked the most pronounced accumulation of MSR transcripts (> 30,000-fold) without eliciting a high level of DNA damage. Cbl0137 destabilizes nucleosomes and induces Z-DNA (supplementary Figure S5) [57], which could reflect a broad collapse of the chromatin structure. Intriguingly, Cbl0137 is the only one of the tested compounds that, in addition to inducing MSR transcript accumulation, also deregulated LINE L1Md_A transcripts (supplementary Figure S6).

To address whether DNA damage per se would induce massive MSR up-regulation, we exposed MEF cells for 24 h with increasing concentrations of the anticancer compounds cisplatin (7 mM), mitomycin C (1 mM) and bleomycin (7 mM). Cisplatin [59] and mitomycin C [60] are DNA alkylating agents that form inter-strand DNA crosslinks and induce DNA lesions by collapsing DNA replication forks. By contrast, bleomycin is a radiomimetic that directly causes double-stranded breaks (DSB) through oxidative cleavage of DNA [61]. We observed γH2A.X signals (DNA damage) for cisplatin and mitomycin C that was further enhanced for bleomycin (Fig. 6D). However, only for bleomycin exposure, but not for cisplatin nor mitomycin C, did we detect accumulation of MSR transcripts that even exceeded the genistein-mediated MSR up-regulation (Fig. 6E). Intriguingly, DSB in heterochromatin have been shown to result in decompaction and unfolding of the heterochromatin structure [54, 55, 62, 63]. Together, these comparative analyses suggest that damaging and possible relaxation of the heterochromatin structure, either by destabilization of nucleosomes (Cbl0137), topoisomerase poisoning (topotecan, genistein, etoposide) or direct induction of DSB (bleomycin) could trigger transcriptional stimulation of MSR sequences.

### Depletion or catalytic inhibition of Top2β modestly increases MSR transcripts

**Fig. 7:**
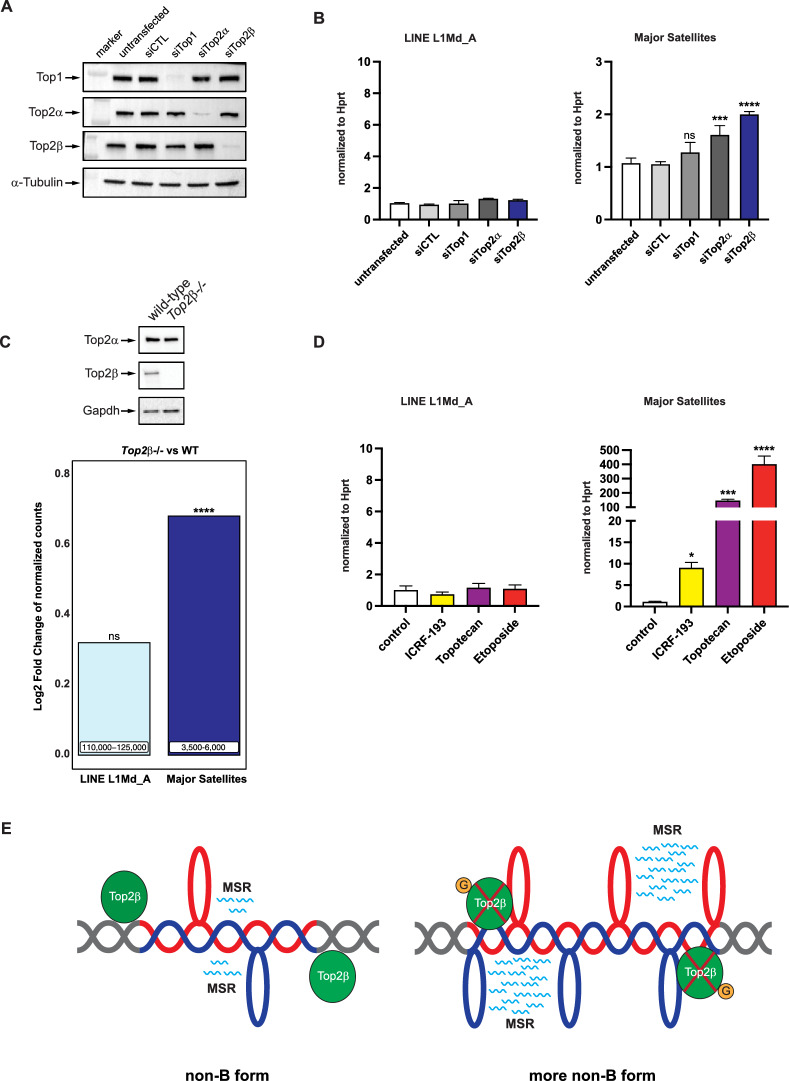
Top2β dysfunction and a topological DNA repeat model for MSR transcription. **A** Western blot for the detection of Top1, Top2α and Top2β in wt MEF cells and in MEF cells that are siRNA-depleted for Top1, Top2α or Top2β. Tubulin expression is used as a loading control. **B** RT-qPCR for LINE L1Md_A (left panel) and MSR (right panel) transcripts from wt MEF cells (untransfected) and MEF cells that are siRNA-depleted for Top1, Top2α and Top2β. Values are normalized to *Hprt*. Asterisks indicate statistically significant differences (***p < 0.001, ****p < 0.0001, ns is not significant, one-way ANOVA, n = 4). **C** Quantification of Log2FoldChanges of normalized counts for LINE L1Md_A and MSR transcripts derived from HiSeq RNA sequencing in wt and *Top2β−/−* MEF cells. The numbers in the bar graphs display the mean number of normalized reads that can be aligned to LINE L1Md_A (110,000–125,000 normalized counts) and MSR (3500–6000 normalized counts) repeat sequences. Asterisks indicate statistically significant differences (****p < 0.0001, FDR/Benjamini-Hochberg, ns is not significant, n = 2). On top, a Western blot verification for the absence of Top2β in the *Top2β−/−* MEF cells is also shown. **D** RT-qPCR for LINE L1Md_A (left panel) and MSR (right panel) transcripts from wt MEF cells (control) and MEF cells that were exposed to ICRF-193, topotecan or etoposide Values are normalized to *Hprt*. Asterisks indicate statistically significant differences (*p < 0.05, ***p < 0.001, ****p < 0.0001, ns is not significant, t-test relative to control, n = 3). **E** Topological model for the genistein-mediated transcriptional stimulation of MSR expression. In this model, reiterated A/T-rich MSR units are intrinsically prone to present a DNA template with partially unwound DNA that is controlled and limited by Top2β. Genistein (G, orange circle) will poison and trap Top2β, which then allows for an accumulation of non-B form DNA exposing more single stranded DNA or R-loops (indicated by the red and blue extensions of the DNA double helix). The non-B form DNA with exposed single-stranded DNA or R-loops could function as a ‘promoter-mimic’ for RNA polymerase II (not shown) engagement to generate basal levels of MSR transcripts (wavy blue lines) or greatly enhanced levels of MSR transcripts when more non-B form DNA accumulates. See discussion for further explanation

Genistein has been reported as a Top2β inhibitor [31, 33] and cells deficient for *Top2β* were shown to be resistant to genistein-induced cell growth inhibition [31]. There are two *Top2* genes in the mouse genome. *Top2β* is expressed throughout the cell cycle with described functions for Top2β in transcriptional regulation, while *Top2α* expression is enriched at the S/G2 phase of the cell cycle where Top2α is involved in assisting DNA replication [64]. We did siRNA-mediated knock-down for either *Top2α* or *Top2β*, and also for *Top1*, in MEF cells (Fig. 7A). RT-qPCR for MSR specific transcripts indicated modest increase of MSR transcripts after knock-down for *Top2α* and *Top2β* but not after knock-down of *Top1* (Fig. 7B). As a control, LINE L1Md_A transcripts were not elevated.

We then used *Top2β*-deficient MEF cells that were derived from *Top2β*-null mice [37]. HiSeq RNA sequencing reveals modest expansion for MSR transcripts (normalized counts), but not for LINE L1Md_A transcripts, when compared to their levels in wt MEF cells (Fig. 7C). These data indicate a significant difference in the magnitude of MSR up-regulation between a pharmacological block (genistein or etoposide) of Top2β vs. genetic depletion of *Top2β*. While described as a Top2β inhibitor, genistein can also trap Top2-DNA cleavage complexes (Top2cc), although to a lesser degree as compared to Top2 poisons [31]. Trapped Top2cc (or trapped Top1cc) are likely to induce more dominant DNA defects (i.e. topoisomerase associated DSB) and altered chromatin configurations, as they would occur with the depletion of Top2 (or Top1) enzymes. We further supported this notion by using ICRF-193, a known catalytic inhibitor (but not poison and does not induce DSB) of Top2β [65]. Catalytic inhibition of Top2β by ICRF-193 (1 mM for 24 h) resulted in a modest, but significant, increase (< 10-fold) of MSR transcripts, which is considerably less than the > 100–300 fold accumulation of MSR transcripts after topotecan or etoposide (Fig. 7D).

## Discussion

In this study, we identified the natural compound genistein as a novel and potent inducer for major satellite repeat (MSR) expression in mouse heterochromatin. The results are consistent with a role for genistein in blocking Top2β and suggest that the transcriptional response of mouse heterochromatin is guided by an altered topology of the underlying A/T-rich MSR DNA repeat arrays. In addition, the data reveal a novel function for genistein in stimulating MSR transcription that may contribute to the growth inhibitory and therapeutic properties of this natural compound.

### A topological DNA repeat model for MSR transcription

We identified genistein as a novel compound that selectively stimulates MSR transcription. There is a massive up-regulation of MSR transcripts (> 100-fold by RT-qPCR) that greatly exceeds the 3- to 5-fold increase of MSR transcripts in *Suv39h* double-null MEF cells [35]. In addition, genistein-mediated up-regulation of MSR transcripts occurs without reducing H3K9me3 or HP1α at MSR chromatin. This indicates that genistein-induced response of MSR expression is primarily directed by stimulating transcriptional activity and not by transcriptional derepression or chromatin desilencing. Indeed, genistein-mediated accumulation of MSR transcripts can be blocked by RNAPII inhibitors or considerably reduced with JQ1, which attenuates Brd4-assisted transcriptional stimulation. While the MSR consensus sequence is A/T-rich, it has no canonical promoter architecture and lacks a TATA box [5]. Several transcriptional start sites (TSS) in the MSR sequence have been mapped, which can initiate bi-directional transcription [10]. However, the majority of MSR RNA is not poly(A)-adenylated and does not contain a 5’cap [14]. These insights suggest that transcriptional regulation and RNA processing of heterochromatic MSR expression differs from the well-defined RNAPII initiation and elongation mechanisms at *bona fide* eukaryotic promoters [66, 67]. While we propose a topological model for RNAPII engagement at MSR DNA that would be enhanced by exposing more unwound DNA (see below), analysis of the full assembly and composition of the RNAPII machinery for heterochromatic MSR expression will require future investigation.

In addition to A/T-richness and a possible topological bias, chromatin alterations do underlie significant derepression of MSR expression, as was recently shown by histone H1 depletion in MEF cells [68]. In human cells, satellite and other repeat sequences are deregulated by curaxin Cbl0137 treatment, which is a described FACT inhibitor inducing global destabilization of nucleosomes [57].

A striking result from our study is the selective response of major satellite repeat transcription to Top1 (topotecan) and Top2 (etoposide) poisons and to genistein. A recent report has shown that catalytical inhibition of Top2 enzymes induces heterochromatin damage and is characterized by increased γH2A.X signals at clustered repetitive elements including MSR sequences [65]. Top2 is known to be a structural component of heterochromatin [69, 70]. The selective response of major satellite repeats to topoisomerase dysfunction, both for transcriptional stimulation (this study) or for increased DNA damage [65] exposes them as preferred genomic targets that are under topological control.

Both Top1 (resolves supercoils) and Top2 (resolves catenates) are essential to release torsional and replicative stress. This will be particularly important, if the underlying DNA is prone to form altered topologies. Major satellite repeats are A/T-rich sequences and have been proposed to have a non-B form DNA conformation [71]. In addition, it was recently shown that major satellite repeats display an altered DNA shape  with narrower DNA minor grooves [72]. These distinct biophysical properties discriminate A/T-rich major satellite repeats from G/C-rich LINE repeats and can help to explain why MSR sequences selectively respond to topoisomerase dysfunction. Our contig analysis of pericentric arrays of mouse MSR repeats indicate ‘head-to-tail’ configurations of the MSR repeat units that are arranged in direct repeat reiterations (see Fig. 2D). Direct tandem repeats have been suggested to form alternative DNA structures, in particular ‘slipped-stranded’ DNA or S-DNA [73]. ‘Slipped-stranded’ DNA results from mispairing of complementary repeats, thereby exposing single-stranded DNA loops, and represents a favorable structure if superhelical DNA is under torsional stress. By contrast, left-handed Z-DNA that is common for many microsatellite sequences and which is induced by curaxin Cbl0137 treatment [57], does not appear to be enriched in genistein-stressed MEF cells (supplementary Figure S5).

MSR repeat transcripts largely remain chromatin associated and analysis of their secondary structure has shown that they maintain unpaired loops of single-stranded RNA which facilitate RNA:DNA hybrid or R-loop formation [14]. While a role for Top2 in relaxing RNA:DNA hybrids has not been described, Top3β is a dual activity type 1A topoisomerase that can catalyze strand passage reactions for both DNA and RNA [74]. Further, depletion of Top1 in human cells increases R-loop formation in gene-poor and repeat-rich regions of the genome [75]. Impairing Top1 and Top2 activities could therefore result in elevated levels of single-stranded DNA or R-loop formation, particularly at DNA repeat regions that are prone to engage in non-B form DNA (see model Fig. 7E). R-loops have been shown to promote antisense transcription [76] and could function as ‘promoter-mimics’ [77] that facilitate RNAPII engagement with non-B form DNA. In addition, Top2α inhibition has recently been shown to release RNAPII pausing [78].

### MSR transcriptional stimulation reveals a novel function for genistein

MSR transcription in mouse fibroblast cells has been shown to be proliferation-dependent and cell cycle regulated, such that there are increased MSR transcript levels at the G1/S boundary and the presence of small (< 200 bp) MSR transcripts in mitotic cells [9]. Northern blot analysis of RNA from genistein-stressed MEF cells reveals a smear of MSR transcripts spanning a broad size range and indicates that a heterogeneous population of MSR transcripts becomes up-regulated. Genistein-mediated MSR transcriptional stimulation requires a cycling G1 cell population and is significantly attenuated when cells are blocked at the G2/M restriction point. A role for genistein in arresting cell cycle progression and in providing growth-inhibitory or apoptotic effects in several human cancer cell lines has been well described [30, 32, 34]. Our data in mouse fibroblasts reveal a novel function for genistein and connect the genistein-mediated up-regulation of heterochromatic MSR expression with increased susceptibility to cell cycle delay, defects in mitotic progression and DNA damage. Since this genistein-mediated MSR transcription requires a cycling G1 cell population and is not effective in post-mitotic cells or blocked G2/M cells, highly proliferating cancer cells may be more vulnerable to genistein exposure. While this potent induction of MSR transcription supports a novel function for genistein as an anticancer compound, genistein-mediated accumulation of MSR transcripts persists at high levels even after the removal of genistein (see Fig. 1E). Prolonged and aberrant up-regulation of satellite repeats are known to provoke recombination defects and genomic instabilities [19–21], both of which drive oncogenic transformation. Thus, dependent on the proliferative status of the target cells, genistein may exert either anticancer or pro-oncogenic activities.

We have recently shown that an altered repeat to gene expression ratio can stratify risk prediction in acute myeloid leukemia in humans and that AML patient subgroups with elevated repeat and satellite RNA expression correlate with a more favorable prognosis [79]. Exposure of human CD34 + T-cells to genistein selectively up-regulates human SatIII (GAATG)n repeats and some ERV sequences (supplementary Figure S7). Curiously, high levels of SatIII RNA in human lung cancer cells have also been suggested to provide etoposide resistance by sequestering TOP2A to nuclear stress bodies [80]. Although the organization and sequence composition of human satellite DNA is different from that of mouse satellite DNA, the function of DNA topoisomerases is conserved. Recently, a threshold limit of > 300-fold up-regulation of MSR transcripts has been shown to break heterochromatin organization and to irreversibly compromise cell viability in MEF cells [81]. While future studies with human cells and with cancer models are required, it is plausible that the dual potential of genistein, both as an anticancer or pro-oncogenic compound, would depend on the magnitude and duration of transcriptional up-regulation of DNA repeat and satellite elements that are under topological control.

## Supplementary Information

Additional file 1.

Additional file 2.

## Data Availability

All data generated or analysed during this study are included in this published article [and its supplementary information files]. The bioinformatic analyses for the MSR Contig assembly will be detailed elsewhere. HiSeq RNA data for untreated and genistein exposed MEF cells have been deposited with the following databases: RNA-Seq data: Gene Expression Omnibus GSE291747. https://www.ncbi.nlm.nih.gov/geo/query/acc.cgi?acc=GSE291747. RNA-Seq data: Gene Expression Omnibus GSE291959. https://www.ncbi.nlm.nih.gov/geo/query/acc.cgi?acc=GSE291959.

## Appendix

### List of compounds

**Table:**

Compound	Catalogue number	Duration	Final concentration
Aphidicolin	A0781 (Sigma Aldrich)	24 h	1 μM, 15 μM
β-Estradiol	E8875 (Sigma Aldrich)	24 h	10 nM
Cbl0137	19110 (Cayman)	24 h	1 μM
Daidzein	D7802 (Sigma Aldrich)	24 h	50 μM
5,6-Dichloro-1-β-d-ribofuranosylbenzimidazole (DRB)	D1916 (Sigma Aldrich)	24 h	50 μM, 100 μM
Etoposide	E1383 (Sigma Aldrich)	24 h	1 μM
Genistein	G6649 (Sigma Aldrich)	3, 24, 48 h	1, 5, 25, 50 μM
H_2_O_2_	H1009 (Sigma Aldrich)	24 h	250 μM
JQ1	SML1524 (Sigma Aldrich)	24 h	1 μM
Rapamycin	R0395 (Sigma Aldrich)	24 h	20 nM
RO-3306	SML0569 (Sigma Aldrich)	24 h	10 μM
Topotecan	T2705 (Sigma Aldrich)	24 h	1 μM
Triptolide	T3652 (Sigma Aldrich)	24 h	0.5 μM, 1 μM
Cisplatin	P4394 (Sigma Aldrich)	24 h	7 μM
ICRF-193	FBM-10–5056 (Biozol)	24 h	1 mM
Mitomycin C	M5353 (Sigma Aldrich)	24 h	1 mM
Bleomycin	B7216 (Sigma Aldrich)	24 h	7 mM

### List of oligonucleotides

**Table:**

Primer set	Sequence 5′ → 3’	Application
5S rRNA	Fw: GTCTACGGCCATACCACCCTG Rv: AGCCTACAGCACCCGGTATTCC	RT-qPCR
Hprt	Fw: AGTGATAGATCCATTCCTATGACTGTAG Rv: GTTAAAGTTGAGAGATCATCTCCACC	RT-qPCR
LINE L1Md_A	Fw: ACTGCGGTACATAGGGAAGC Rv: TGTGATCCACTCACCAGAGG	RT-qPCR, ChIP-qPCR
Major Satellites (MSR)	Rep1c Fw: TGGAATATGGCGAGAAAACTG Rep1c Rv: AGGTCCTTCAGTGGGCATTT	RT-qPCR, ChIP-qPCR
β-Actin promoter	Fw: TTTTATGGCTCGAGTGGCCG Rv: CTGCAAAGAAGCTGTGCTCG	ChIP-qPCR
Zfp180 Exon 5	Fw: CCGTACAGGTGCAATCTGTG Rv: GTTTGTAGCTCTGGCGGAAC	ChIP-qPCR
MSR-SR4 probe	Rv: GATTTCGTCATTTTTCAAGTCGTCAAGTGGATGTTTCTCAT	NB

### List of RNA-FISH LNA probes

**Table:**

Target	Sequence 5′ → 3’	
LNA oligos for RNA-FISH (capital letters indicate locked nucleic acids)		
	Forward strand detection	Reverse strand detection
oMajL_1	bio-gTcCtAcAgTgGaCaTtTcTaAaT	bio-AtTtAgAaAtGtCcAcTgTaGgAc
oMajL_2	bio-atTtTcAgTtTtCcAtAtT	bio-AaTaTgGcAaGaAaAcTgAaAat
oMajL_3	bio-agTcGtCaAgTgGaTgTtTcTcAtT	bio-AaTgAgAaAcAtCcAcTtGaCgAct
oMajL_4	bio-CaGtGtGcAtTtCtCaTtTtTcA	bio-TgAaAaAtGaGaAaTgCaCaCtg

### List of primary antibodies

**Table:**

Primary antibody	Catalogue number	Application
53bp1	Ab36823 (Abcam)	IF
γH2A.X	05-636 (Millipore)	WB, IF
Gapdh	Ab8245 (Abcam)	WB
α-Tubulin	Ab4074 (Abcam)	WB
H3	Ab1926 (Abcam)	WB
H3K9me3	Am39161 (Active Motif)	ChIP
H3K9me3	Ab8898 (Abcam)	IF
H4K20me3	Jenuwein Lab (#0083)	IF
Rpa2	2208 (Cell Signaling)	IF
Hp1α	Ab109028 (Abcam)	IF
Hp1α	2616 (Cell Signaling)	ChIP
RNA Pol 2 (Rpb1)	14958 (Cell Signaling)	ChIP
Top1	Ab85038 (Abcam)	WB
Top2α	Ab52934 (Abcam)	WB
Top2β	Ab264158 (Abcam)	WB
Z-DNA	NB100-749 (Novus Biologicals)	IF

### List of secondary antibodies for IF and WB

**Table:**

Secondary antibody	Catalogue number	Application
Goat α-Mouse Cy3	A10521 (Invitrogen)	IF
Goat α-Rabbit Cy5	A10523 (Invitrogen)	IF
Goat α-Rat Cy3	A10522 (Invitrogen)	IF
Donkey α-Sheep Alexa 488	713-545-147 (Jackson Labs)	IF
Goat α-Rabbit HRP	111-035-144 (Jackson Labs)	WB
Rabbit α-Mouse HRP	315-035-006 (Jackson Labs)	WB

### List of siRNA (Dharmacon)

**Table:**

siRNA target	Cat. Nr	Final concentration
Top1	L-047567-01-0005	37.5 nM
Top2α	L-043916-01-0005	37.5 nM
Top2β	L-042134-00-0005	37.5 nM
