# Supplementary material for: The isoflavone genistein selectively stimulates major satellite repeat transcription in mouse heterochromatin
Source: Epigenetics Chromatin. 2025 Aug 25;18:58. doi: 10.1186/s13072-025-00623-4 (PMC12376718; doi:10.1186/s13072-025-00623-4)
Supplement: Supplementary file 1 — Additional file 1. [file 13072_2025_623_MOESM1_ESM.pdf]

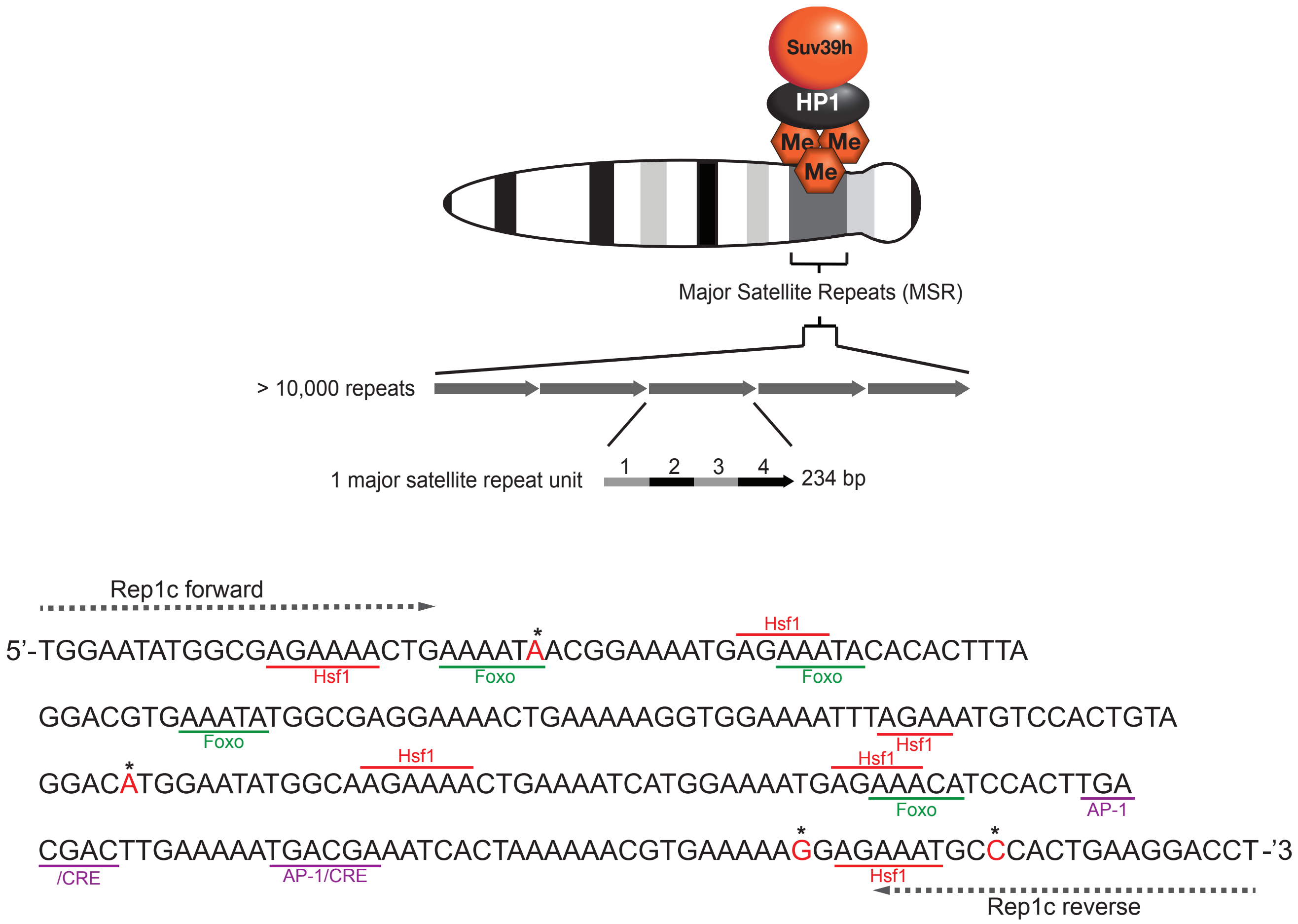
Suv39h
HP1
Me
Me
Me
Major Satellite Repeats (MSR)
> 10,000 repeats
1 major satellite repeat unit
1
2
3
4
234 bp
Rep1c forward
5'-TGGAATATGGCGAGAAAACTGAAAATAACGGAAAATGAGAAATACACACTTTA
GGACGTGAAATATGGCGAGGAAAACTGAAAAAGGTGGAAAATTTAGAAATGTCCACTGTA
GGACATGGAATATGGCAAGAAAACTGAAAATCATGGAAAATGAGAAACATCCACTTGA
CGACTTGAAAAATGACGAAATCACTAAAAAACGTGAAAAAGGAGAAATGCCCACTGAAGGACCT-'3
Hsf1
Foxo
AP-1
/CRE
AP-1/CRE
Rep1c reverse

Figure S1

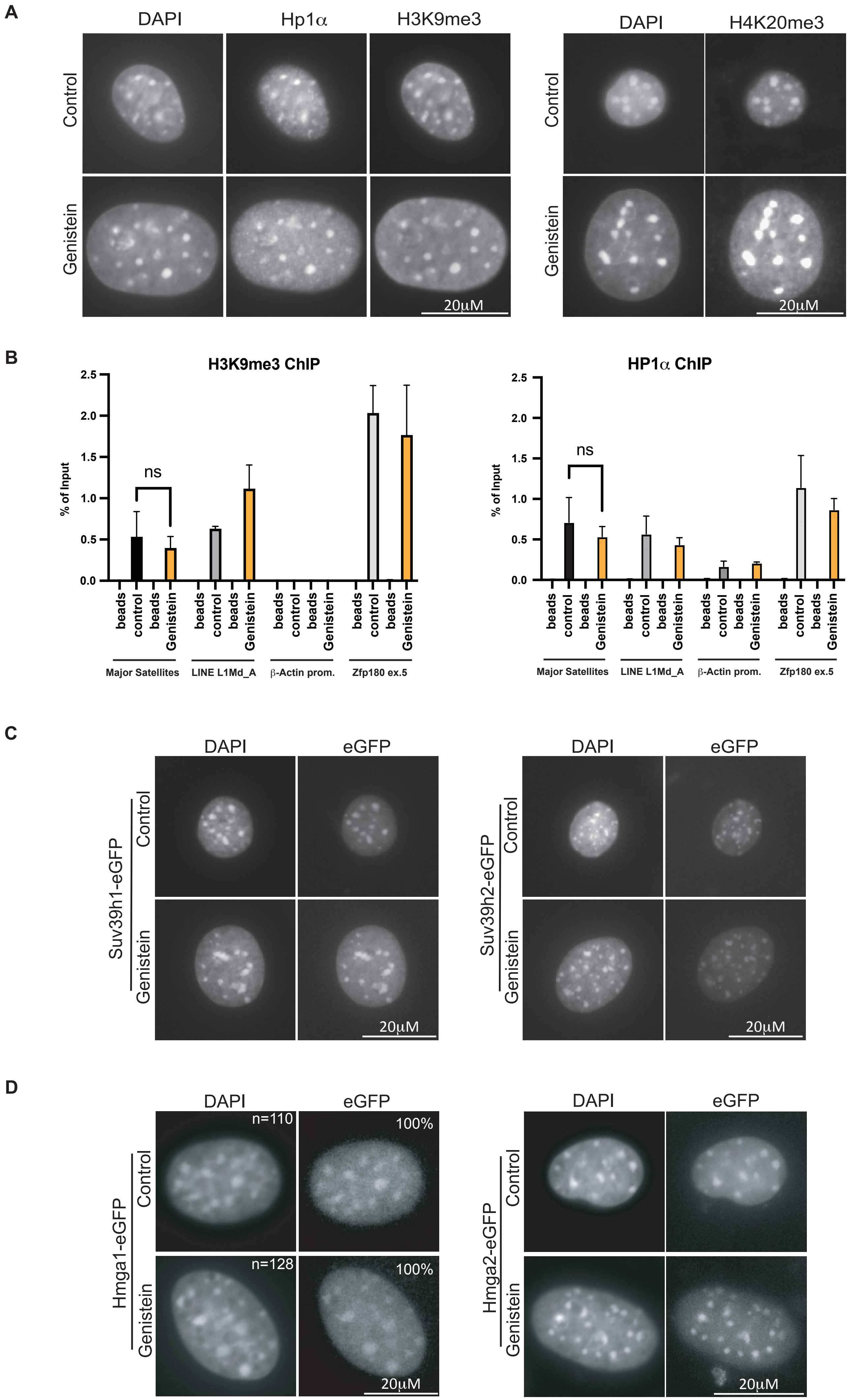
A
DAPI
Hp1α
H3K9me3
DAPI
H4K20me3
Control
Genistein
20μM
B
H3K9me3 ChIP
HP1α ChIP
% of Input
ns
beads
control
Genistein
Major Satellites
LINE L1Md_A
β-Actin prom.
Zfp180 ex.5
C
DAPI
eGFP
Suv39h1-eGFP
Suv39h2-eGFP
Control
Genistein
20μM
D
DAPI
eGFP
Hmga1-eGFP
Hmga2-eGFP
n=110
n=128
100%
Control
Genistein
20μM

Figure S2

A

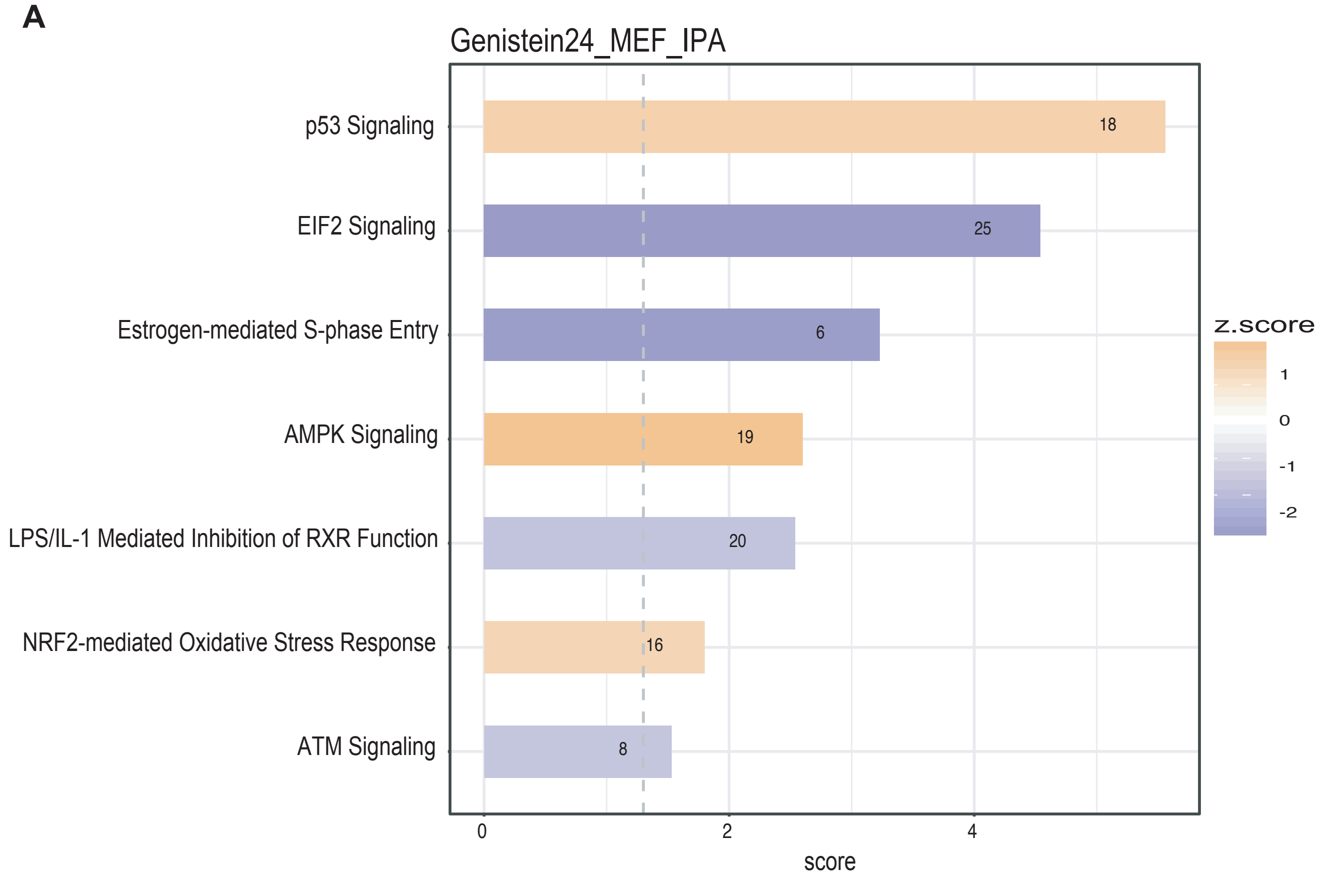

Genistein24_MEF_IPA
p53 Signaling
18
EIF2 Signaling
25
Estrogen-mediated S-phase Entry
6
AMPK Signaling
19
LPS/IL-1 Mediated Inhibition of RXR Function
20
NRF2-mediated Oxidative Stress Response
16
ATM Signaling
8
0
2
4
score
z.score
1
0
-1
-2

B

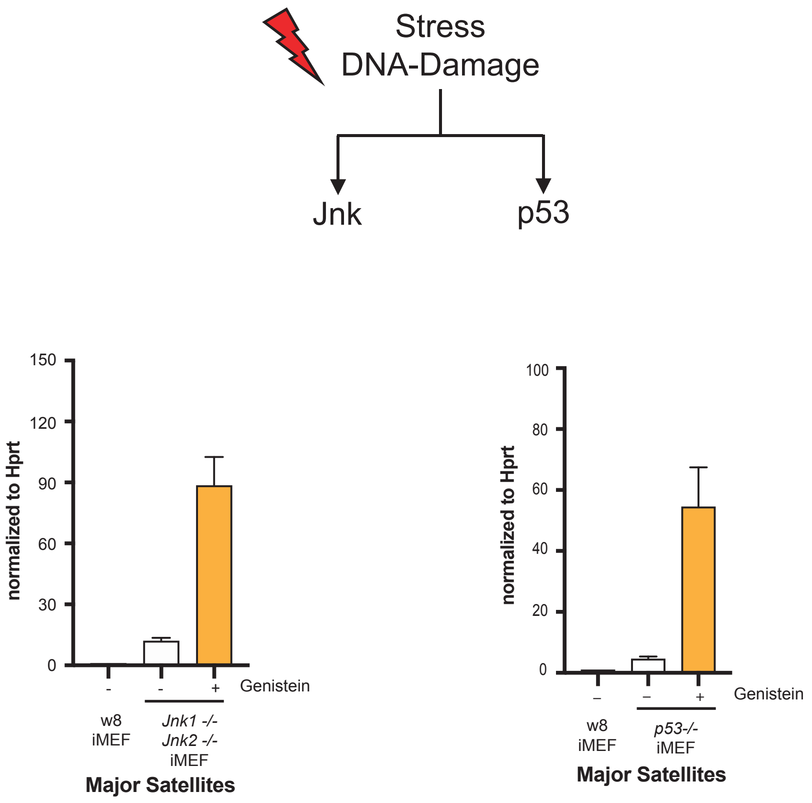

Stress
DNA-Damage
Jnk
p53
150
120
90
60
30
0
normalized to Hprt
-
-
+
Genistein
w8
iMEF
Jnk1 -/-
Jnk2 -/-
iMEF
Major Satellites
100
80
60
40
20
0
normalized to Hprt
–
–
+
Genistein
w8
iMEF
p53-/-
iMEF
Major Satellites

Figure S3

A

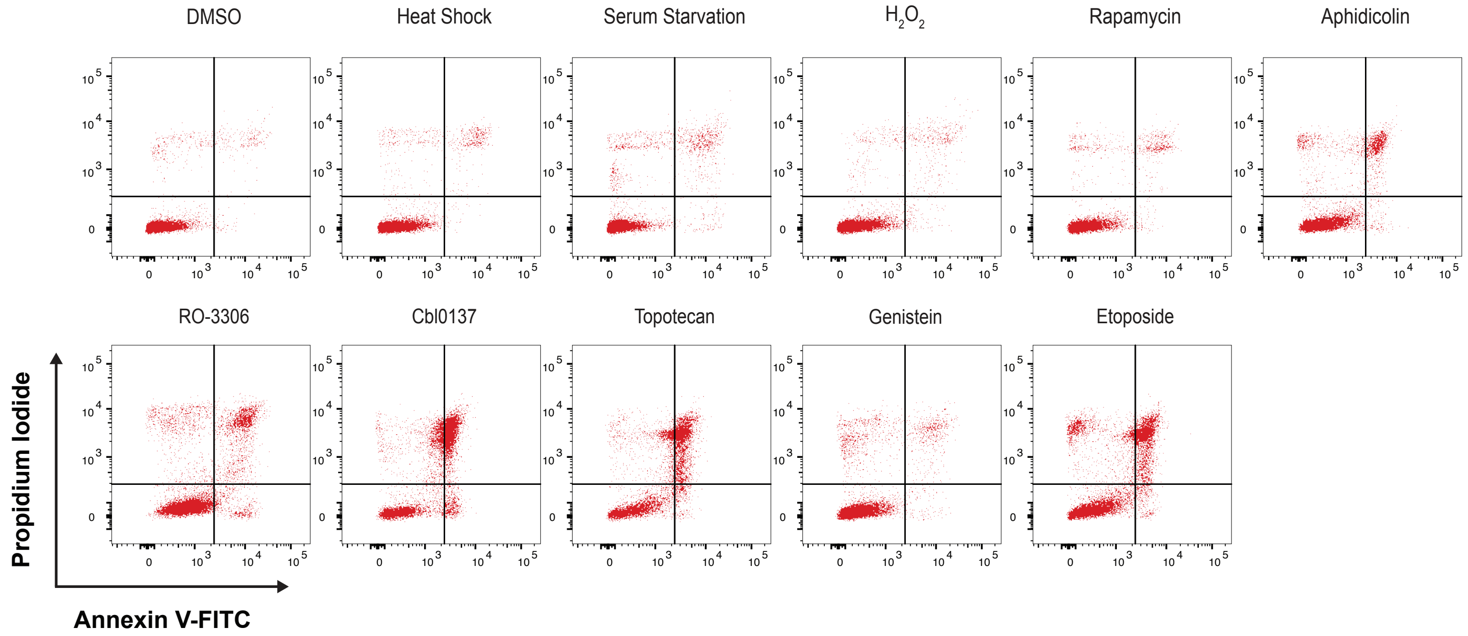
DMSO
Heat Shock
Serum Starvation
H_2O_2
Rapamycin
Aphidicolin
RO-3306
Cbl0137
Topotecan
Genistein
Etoposide
Propidium Iodide
Annexin V-FITC

B

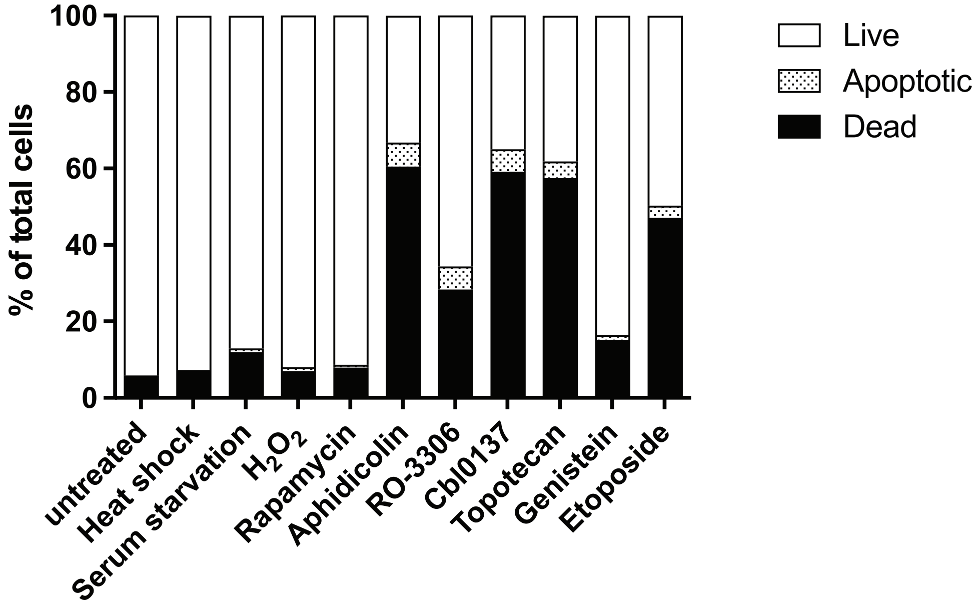
% of total cells
100
80
60
40
20
0
untreated
Heat shock
Serum starvation
H_2O_2
Rapamycin
Aphidicolin
RO-3306
Cbl0137
Topotecan
Genistein
Etoposide
Live
Apoptotic
Dead

Figure S4

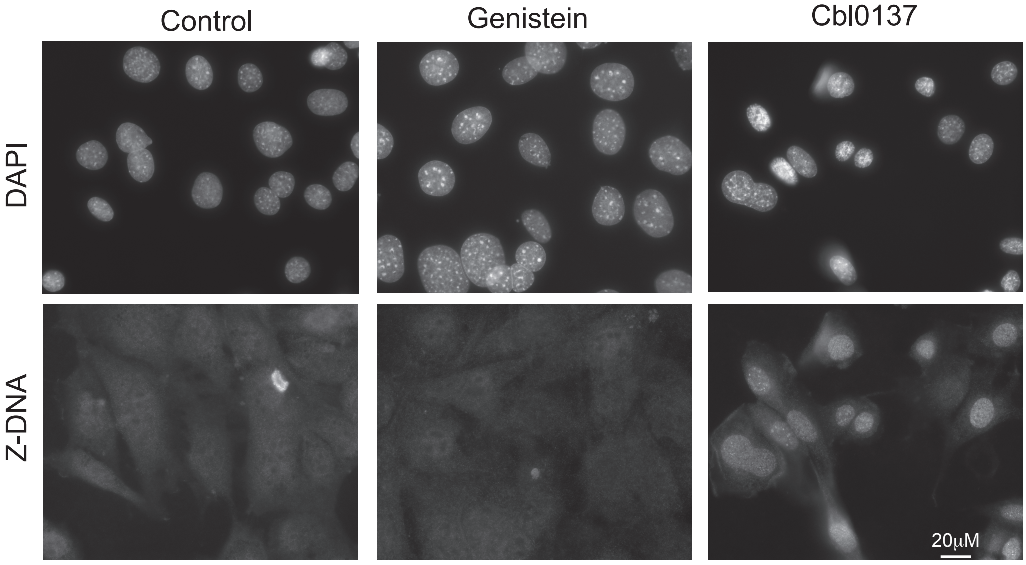

Figure S5

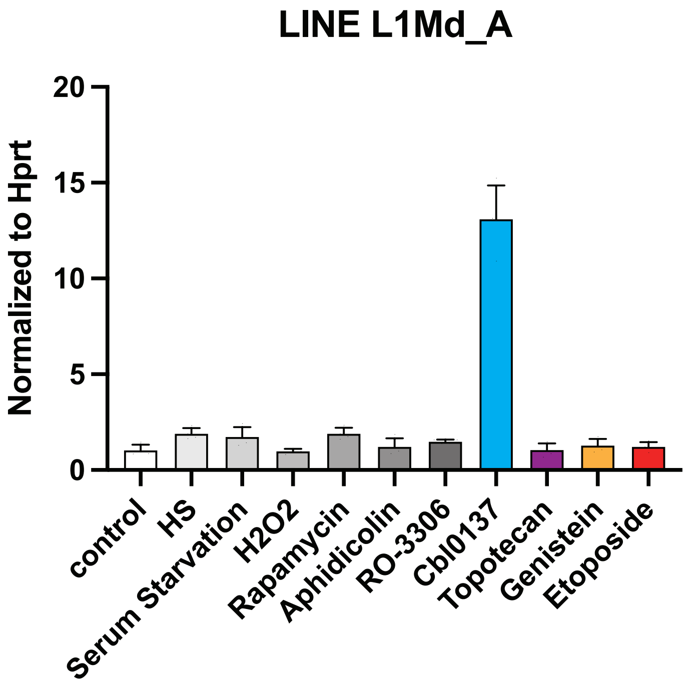
LINE L1Md_A
Normalized to Hprt
20
15
10
5
0
control
HS
Serum Starvation
H2O2
Rapamycin
Aphidicolin
RO-3306
Cbl0137
Topotecan
Genistein
Etoposide

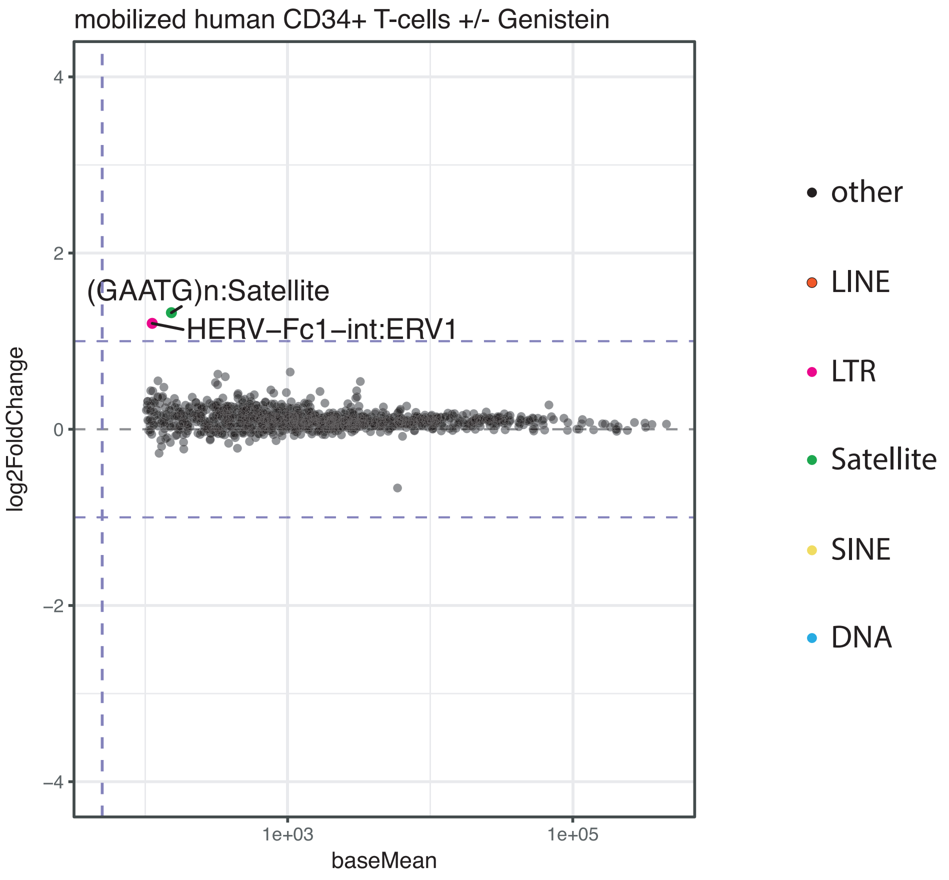
mobilized human CD34+ T-cells +/- Genistein
(GAATG)n:Satellite
HERV–Fc1–int:ERV1
log2FoldChange
4
2
0
−2
−4
1e+03
1e+05
baseMean
other
LINE
LTR
Satellite
SINE
DNA

Figure S7
