# Supplementary material for: The isoflavone genistein selectively stimulates major satellite repeat transcription in mouse heterochromatin
Source: Epigenetics Chromatin. 2025 Aug 25;18:58. doi: 10.1186/s13072-025-00623-4 (PMC12376718; doi:10.1186/s13072-025-00623-4)
Supplement: Supplementary file 2 — Additional file 2. [file 13072_2025_623_MOESM2_ESM.docx]

**SUPPLEMENTAL METHODS**

**Generation of D5-Suv39h-eGFP MEF cells**

*Suv39h* double-null (D5) MEF cells (1) were stably transfected using the Xfect reagent (Clontech) with plasmids (pCAGGS-eGFP-IRES-puro) expressing full-length mouse Suv39h1 or full-length mouse Suv39h2 as eGFP fusion proteins. D5-Suv39h1-eGFP and D5-Suv39h2-eGFP mass cell populations were maintained under puromycin selection. Direct fluorescence for eGFP signal was carried out on an Apotome 2 (Zeiss) confocal microscope. Zen 2 (Zeiss) imaging software was used to collect images.

**Generation of NIH-3T3 cells lines expressing Hmga1-eGFP and Hmga2-eGFP**

NIH-3T3 cells expressing eGFP fusion proteins of Hmga1 and Hmga2 were previously generated in the laboratory using a gene trap screen for heterochromatin components (2). Direct fluorescence for eGFP signal was carried out on an Apotome 2 (Zeiss) confocal microscope. Zen 2 (Zeiss) imaging software was used to collect images.

***Jnk* and *p53* null MEF cells**

*Jnk1/Jnk2* single KO and double KO MEF cells (3) were a kind gift from E. Wagner and L. Bakiri (MedUni, Vienna). *p53^-/-^* null MEF cells (4) were kindly provided by A. Jackson (MRC, Edinburgh).

**Mobilized human CD34+ T-cells**

Mobilized human CD34+ T-cells were purchased from the Heimfeld Lab (Fred Hutchinson Cancer Research Centre, Seattle). Mobilized CD34+ cells were seeded at 2x10^5^ cells/ml and cultured in a flask for a maximum of 6 days in StemSpan™ SFEM II (Stem Cell Technologies, #09655) with 1% Stem Span CC100 cytokine cocktail (Stem Cell Technologies, #02690) and 2% Penicillin-Streptomycin (Sigma Aldrich, P4333). Mobilized CD34+ cells were grown without agitation at 37°C in 5% CO_2_. Bioinformatic analysis of repeat element expression in untreated (DMSO alone) or genistein-exposed (24 hrs with 75 μM genistein) mobilized CD34+ cells was done as described for repeat element expression in human AML CD34+ cells (5).

**Chromatin immunoprecipitation (ChIP)**

ChIP was performed as previously performed (6). For single cross-linking ChIP, cells were washed with PBS and incubated with 1% formaldehyde (Sigma) at RT for 15 min. The reaction was quenched by adding 1:20 volume of 2.5 M glycine (Sigma) for 5 min and chromatin was fragmented by sonication. Antibodies for single cross-linking ChIP were: 4 μg of anti-H3K9me3 (Abcam, ab8898).

For double cross-linking ChIP, cells were washed with PBS and incubated with 2 mM DSG (ChemScene, 79642-50-5) at RT for 45 min. Cells were washed twice with PBS and processed by 1% formaldehyde fixation (15 min at RT) and glycine quenching. Chromatin was fragmented at 4°C to an average size of 250 bp using a COVARIS S220 focused-ultrasonicator. For each ChIP reaction, 10 μg of sonicated chromatin was incubated with antibody on a rotating wheel at 4°C O/N and captured with magnetic Protein G Dynabeads (Thermo Fisher, 10004D). Beads were washed and eluted in EB buffer (1% SDS and 0.1 M NaHCO3) and incubated in the presence of 50 μg RNase A (Thermo Fisher Scientific, EN0531) and 60 μg proteinase K (Thermo Fisher Scientific, EO0491) at 37°C for 1 hrs, followed by additional incubation at 65°C O/N in a Thermomixer (Eppendorf). Decross-linked DNA was purified using a PCR purification kit (Macherey-Nagel, 740609.250). Antibodies for double cross-linking ChIP were: 2.5 μg of anti-HP1α (Cell Signaling, 2616) and 2.7μg of anti-Rpb1 (RNA Pol II, Cell Signaling, 14958).

**SUPPLEMENTAL REFERENCES**

1. Peters AH, O'Carroll D, Scherthan H, Mechtler K, Sauer S, Schofer C, Weipoltshammer K, Pagani M, Lachner M, Kohlmaier A, Opravil S, Doyle M, Sibilia M, Jenuwein T. Loss of the Suv39h histone methyltransferases impairs mammalian heterochromatin and genome stability. Cell. 2001;107(3):323-37.

2. Fodor BD, Shukeir N, Reuter G, Jenuwein T. Mammalian Su(var) genes in chromatin control. Annu Rev Cell Dev Biol. 2010;26:471-501.

3. Sabapathy K, Hochedlinger K, Nam SY, Bauer A, Karin M, Wagner EF. Distinct roles for JNK1 and JNK2 in regulating JNK activity and c-Jun-dependent cell proliferation. Mol Cell. 2004;15(5):713-25.

4. Reijns MA, Rabe B, Rigby RE, Mill P, Astell KR, Lettice LA, Boyle S, Leitch A, Keighren M, Kilanowski F, Devenney PS, Sexton D, Grimes G, Holt IJ, Hill RE, Taylor MS, Lawson KA, Dorin JR, Jackson AP. Enzymatic removal of ribonucleotides from DNA is essential for mammalian genome integrity and development. Cell. 2012;149(5):1008-22.

5. Onishi-Seebacher M, Erikson G, Sawitzki Z, Ryan D, Greve G, Lubbert M, Jenuwein T. Repeat to gene expression ratios in leukemic blast cells can stratify risk prediction in acute myeloid leukemia. BMC Med Genomics. 2021;14(1):166.

6. Bulut-Karslioglu A, De La Rosa-Velazquez IA, Ramirez F, Barenboim M, Onishi-Seebacher M, Arand J, Galan C, Winter GE, Engist B, Gerle B, O'Sullivan RJ, Martens JH, Walter J, Manke T, Lachner M, Jenuwein T. Suv39h-dependent H3K9me3 marks intact retrotransposons and silences LINE elements in mouse embryonic stem cells. Mol Cell. 2014;55(2):277-90.

Supplementary Figure S1: **Chromosome ideogram of mouse pericentric heterochromatin and DNA sequence of the consensus unit (234bp) of a major satellite repeat.**

The pericentric regions of mouse chromosomes are decorated by Suv39h-mediated H3K9me3 and association of HP1. The underlying DNA is characterized by large arrays of reiterated copies of major satellite repeats (MSR). The DNA sequence (forward strand) of one unit of a MSR consensus, specifying the 4 subrepeats is shown. The displayed MSR sequence (MSR Rep1c) is derived from sequencing analyses in mESC and MEF cells and differs by four nucleotide changes (indicated in red and with a small asterisk) from the mouse MSR consensus sequence. Also highlighted are described transcription factor binding sites for stress-response transcription factors (Hsf1, Foxo and AP1/CRE). MSR Rep1c forward and MSR Rep1c reverse primers used for RT-qPCR are indicated by the dashed lines.

Supplementary Figure S2: **Analysis for heterochromatin components in mouse fibroblasts cells exposed to genistein.**

A) Immunofluorescence for the localization of HP1α and H3K9me3 (left panel) or H4K20me3 (right panel) in untreated or genistein-exposed MEF cells. For each sample, n≥110 cells were analyzed. B) ChIP-qPCR to detect H3K9me3 and HP1α at major satellite and LINE L1Md-A repeats and at control sequences (β-actin promoter and Zfp180 exon 5) in untreated or genistein-exposed MEF cells. Error bars reflect the standard deviation from n=3 biological replicates. C) Fluorescence microscopy for the localization of Suv39h1-eGFP and Suv39h2-eGFP in D5-Suv39h1-EGFP and D5-Suv39h2-EGFP MEF cells (see supplementary methods), either untreated or exposed to genistein. For each sample, n≥59 cells were analyzed. D) Fluorescence microscopy for the localization of Hmga1-eGFP and Hmga2-eGFP in mouse fibroblast cells, either untreated or exposed to genistein (n>110). For each sample, n≥110 cells were analyzed. For all samples, nuclei were counterstained with DAPI. Scale bar is 20 μm.

Supplementary Figure S3: **IPA analysis and stress (Jnk and p53 signaling) response in MEF cells exposed to genistein.**

A) Ingenuity pathway analysis (IPA) derived from HiSeq RNA libraries of untreated and genistein-exposed MEF cells (n=2). Displayed are the top 7 signaling pathways that are dysregulated in genistein-exposed MEF cells. B) RT-qPCR for MSR transcripts in *Jnk1/Jnk2* double-null MEF cells (left panel) or *p53*-null MEF cells (right panel), either untreated or exposed to genistein. Values are normalized to *Hprt*. Error bars reflect the standard deviation from n=3 biological replicates.

Supplementary Figure S4: **Apoptotic indices of MEF cells exposed to various stress conditions.**

A) FACS profiles (propidium iodide and Annexin V-FITC staining) of MEF cells exposed to stress signals (heat shock, H_2_O_2_, serum starvation), cell cycle blocks (rapamycin, aphidicolin, RO-3306), chromatin damage (Cbl0137) and topoisomerase poisoning (topotecan, genistein, etoposide). B) Quantification of live, apoptotic and dead cells is represented by stacked bar graphs.

Supplementary Figure S5: **Immunofluorescence analysis for Z-DNA in MEF cells, either untreated or exposed to genistein or curaxin (Cbl0137).**

For each sample, n≥116 cells were analyzed. Nuclei were counterstained with DAPI. Scale bar is 20 μm.

Supplementary Figure S6: **Analysis of LINE L1Md-A expression after exposure of MEF cells to various stress signals.**

RT-qPCR for LINE L1Md-A transcripts in MEF cells exposed to various stress signals (heat shock, H_2_O_2_, serum starvation), cell cycle blocks (rapamycin, aphidicolin, RO-3306), chromatin damage (Cbl0137) and topoisomerase poisoning (topotecan, genistein, etoposide). Values are normalized to *Hprt*. Error bars reflect the standard deviation from n=3 biological replicates.

Supplementary Figure S7: **Derepression of SatIII (GAATG)n repeat transcripts in genistein-exposed human CD34+ T-cells.**

MA plot for repeat element expression (LINE, LTR-ERV, SINE and satellite repeat classes) in HiSeq RNA libraries from untreated and genistein-exposed (24 hrs with 75 μM genistein) human CD34+ T-cells (n=2). Dashed lines in the MA plot discriminate non-significant expression differences that are below the cut-off (baseMean > 100, log2FoldChange >1 or <-1). (GAATG)n:Satellite denotes human SatIII repeat RNA.
